# TRIM33 loss in multiple myeloma is associated with genomic instability and sensitivity to PARP inhibitors

**DOI:** 10.1038/s41598-024-58828-8

**Published:** 2024-04-16

**Authors:** Roisin M. McAvera, Jonathan J. Morgan, Ana B. Herrero, Ken I. Mills, Lisa J. Crawford

**Affiliations:** 1 Patrick G Johnston Centre for Cancer Research, Queen’s University Belfast, Belfast, BT9 7BL UK; 2 Institute of Biomedical Research of Salamanca (IBSAL), Salamanca, Spain; 3 Molecular Medicine Unit, Department of Medicine, University of Salamanca, Salamanca, Spain; 4 Cancer Research Center-IBMCC (USAL-CSIC), Salamanca, Spain

**Keywords:** Multiple myeloma, DNA damage response, Genomic instability, TRIM33, PARP, Myeloma, Experimental models of disease

## Abstract

Deletions of chromosome 1p (del(1p)) are a recurrent genomic aberration associated with poor outcome in Multiple myeloma (MM.) TRIM33, an E3 ligase and transcriptional co-repressor, is located within a commonly deleted region at 1p13.2. TRIM33 is reported to play a role in the regulation of mitosis and PARP-dependent DNA damage response (DDR), both of which are important for maintenance of genome stability. Here, we demonstrate that MM patients with loss of TRIM33 exhibit increased chromosomal instability and poor outcome. Through knockdown studies, we show that TRIM33 loss induces a DDR defect, leading to accumulation of DNA double strand breaks (DSBs) and slower DNA repair kinetics, along with reduced efficiency of non-homologous end joining (NHEJ). Furthermore, TRIM33 loss results in dysregulated ubiquitination of ALC1, an important regulator of response to PARP inhibition. We show that TRIM33 knockdown sensitizes MM cells to the PARP inhibitor Olaparib, and this is synergistic with the standard of care therapy bortezomib, even in co-culture with bone marrow stromal cells (BMSCs). These findings suggest that TRIM33 loss contributes to the pathogenesis of high-risk MM and that this may be therapeutically exploited through the use of PARP inhibitors.

## Introduction

Multiple Myeloma (MM) is a haematological malignancy characterised by the clonal proliferation of aberrant plasma cells in the bone marrow. Despite significant therapeutic improvements in recent years, MM remains incurable with a 5-year survival rate of approximately 50%. Genomic instability is a hallmark of MM, with almost all patients displaying cytogenetic abnormalities including ploidy, structural rearrangements and copy number abnormalities^1^. Deletion of chromosome 1p (del(1p)), comprising whole arm deletions or interstitial deletions spanning a region from 1p12 to 1p33, are present in up to 30% of MM cases^2^ and associated with high-risk disease^3,4^. Several tumour suppressor genes on 1p, such as CDKN2C and FAM46C, have been associated with disease biology and adverse survival in MM^5–7^ and it is likely that other genes in this region also contribute to the malignant phenotype. The E3 ligase tripartite motif-33 (TRIM33)/ transcriptional intermediary factor-1γ (TIF1γ), located at 1p13.2, was previously identified as part of a 70-gene signature associated with high-risk MM^3^, however, it’s function has not been investigated.

TRIM33 is a member of the TIF1 family of chromatin-binding proteins alongside TRIM24, TRIM28, and TRIM66. The TIF1 proteins are characterised by a conserved N-terminal RBCC (RING domain, two B-box domains, coiled-coil domain) motif that mediates ubiquitination and a C-terminal plant homeodomain (PHD) and bromodomain which recognise histone methylation and acetylation, respectively^8^. TRIM33 has been implicated in many cellular processes including regulation of haematopoiesis^9–11^, embryonic development^12^, immunity^13,14^, mitosis^15,16^ and DNA repair^17–19^. While the function of TRIM33 in cancer has not been fully elucidated, it has been identified as a tumour suppressor in several cancer types, including chronic myelomonocytic leukaemia (CMML) and hepatocellular cancer, where TRIM33 is downregulated through hypermethylation of its promoter region^20,21^. Furthermore, low TRIM33 expression across several cancer types correlates with an increased rate of genomic rearrangements, suggesting a role for TRIM33 in regulating genomic stability^8,16^.

Deficiencies in the DNA damage response (DDR) lead to genomic instability and are a key hallmark of cancer cells. TRIM33 was first identified to participate in the DDR through its interaction with chromatin remodelling enzyme amplified in liver cancer-1 (ALC1)^17^. TRIM33 was found to be rapidly recruited to sites of DNA damage in a PARP-dependent manner, to ensure the timely removal of ALC1. Knockdown of TRIM33 led to retention of ALC1 at DNA damage sites and sensitized cells to the DNA damaging agent bleomycin C. More recent studies utilising large genetic-based screens of bromodomain proteins also indicate a role for TRIM33 in the DDR, demonstrating that TRIM33 localises to DNA lesions and TRIM33 knockdown results in accumulation of γH2AX and micronuclei^18,19^.

Here, we identify a subset of MM patients who have copy number loss of TRIM33, associated with poor prognosis and increased chromosomal instability. We show that shRNA knockdown of TRIM33 in MM cell lines results in accumulation of endogenous DNA damage and leads to slower DNA repair kinetics. Finally, we demonstrate that DDR defects associated with TRIM33 loss can be therapeutically exploited using the PARP inhibitor Olaparib.

## Materials and methods

### Cell lines and reagents

JJN3 and U266 cell lines were purchased from DSMZ, and HS-5 and 293T cell lines were purchased from ATCC. Cells were cultured in a humidified incubator at 37 °C and 5% CO_2_ in RPMI 1640 medium supplemented with 10% foetal bovine serum (FBS), 100 U/mL penicillin and 100 µg/mL streptomycin (Fisher Scientific, Loughborough, UK). Cells were routinely tested for mycoplasma, and authentication using short tandem repeat profiling (STR) was conducted by Eurofins Cell Line Authentication Service (Ebersberg, Germany). Olaparib (S1060) and bortezomib (S1013) stocks were purchased from Selleckchem (Absource Diagnostics Munchen, Germany), resuspended in DMSO and stored at −80 °C.

### Lentiviral transfection

E.coli glycerol stocks of pGIPZ lentiviral shRNA constructs against TRIM33 or a non-targeting control (NTC) were purchased (shRNA listed below) from Dharmacon (Horizon Discovery Ltd., Cambridge, UK). Plasmid DNA was obtained and was transfected into 293T cells to enable production of the lentiviral vectors. 24 h following transfection, the media was refreshed and another 24 h later the virus-containing medium was harvested and filtered (0.45 µm) before being supplemented with polybrene (6 µg/mL) and used to transduce MM cell lines. Antibiotic selection was performed using puromycin (0.5 µg/mL for U266 cells and 1 µg/mL for JJN3 cells) for at least a week, and cells were checked for expression of TurboGFP reporter gene.

TRIM33 shRNA: TTATCTAAACTACTTGAGC.

### Antibodies

The following primary antibodies were used: TRIM33 (#90,051, CST, Leiden, The Netherlands), pCHK1^Ser345^ (#2348, CST), CHK1 (PA5-96,749, Invitrogen), pKAP1^Ser824^ (A300-767A, Bethyl Laboratories), KAP1 (A700-014, Bethyl Laboratories, Cambridge, UK), γH2AX (SAB5600038, Sigma Aldrich, Dorset, UK), GAPDH (ab8245, Abcam, Cambridge, UK), ALC1 (av51324, Abcam), K48-linkage specific polyubiquitination (#8081, CST) and 53BP1 (sc-517281, Santa Cruz Biotechnology, Texas, USA). The following secondary antibodies were used for Western blotting, Anti-Mouse IgG HRP-linked (#7076P2, CST) and Anti-Rabbit IgG HRP-linked (#7074P2, CST), and Anti-Mouse IgG Alexa Fluor 568 (A-11004, ThermoFisher Scientific) was used for immunofluorescence staining. Ubiquitinated proteins were isolated using UbiQapture-Q (Enzo Life Sciences, Exeter, UK) to bind both mono- and poly-ubiquitinated proteins independent of lysine chain linkage.

### Western blotting

At least 1 × 10^6^ cells were harvested and lysed using radioimmunoprecipitation (RIPA) buffer supplemented with protease and phosphatase inhibitors to obtain whole cell extracts. Protein was quantified using the Pierce™ BCA Protein Assay Kit (ThermoFisher Scientific). Equal amounts of protein were denatured in LDS sample buffer (Invitrogen Ltd., Paisley, UK) at 95 °C for 5 min and resolved by SDS-PAGE on 10% Bis–Tris gels (Invitrogen Ltd., Paisley, UK). The protein was transferred to a polyvinylidene fluoride (PVDF) membrane and immunoblotting performed using the indicated antibodies. Densitometric measurements of bands on Western blots were carried out using ImageJ software. Western blot images were inverted, and mean intensity was measured. This was then normalized to the background intensity, and normalized values were plotted.

### Histone extraction

The Histone Extraction kit (ab113476) was used to identify histone binding proteins during selected conditions. Cells were harvested by centrifugation at 1000 rpm for 5 min at 4 °C, resuspended in 1X Pre-Lysis Buffer at 10^7^ cells/ml and lysed on ice with gentle stirring for 10 min. Samples were then centrifuged at 3000 rpm for 5 min at 4 °C and the supernatant removed. Cell pellets were resuspended in lysis buffer, incubated on ice for 30 min and centrifuged at 12,000 rpm for 5 min at 4 °C before the supernatant was transferred to clean Eppendorf tubes.

### Immunofluorescent staining

1 × 10^4^ cells were spun onto slides using a Cytospin 3 centrifuge (ThermoFisher Scientific) and immediately cells were fixed with 4% paraformaldehyde on ice. Cells were permeabilised using 0.4% Triton-X100 and blocked in 3% Bovine Serum Albumin (BSA). Cells were stained using indicated primary and secondary antibodies before addition of 5 μL of DAPI ProLong™ Gold Antifade Mountant (ThermoFisher Scientific). Slides were visualised and representative images acquired the following day using a Nikon Inverted Fluorescence microscope. The number of foci per cell was counted for a minimum of 100 cells per condition and sample names were blinded during this process.

### Colony formation assay

1 × 10^3^ JJN3 cells or 5 × 10^3^ U266 cells were plated in 1 mL of Methocult™ H4230 methylcellulose semi-solid media (Stem Cell Technologies™, Cambridge, UK) and treated with 1 µL of DMSO or Olaparib stock to achieve desired concentration. Plates were left in a humidified incubator at 37°C for 10 days and colonies were stained with Iodonitrotetrazolium Chloride (INT; Sigma Aldrich) at a concentration of 8 mg/mL. Colonies were counted using a light microscope.

### Cell assays

Cell proliferation in co-culture experiments was assessed using CyQUANT™ Direct Cell Proliferation assay (ThermoFisher Scientific) and cytotoxicity was assessed using the CellTox™ Green Cytotoxicity assay (Promega) according to manufacturer’s instructions.

### Co-culture assay

MM cell lines and the bone marrow stromal cell (BMSC) line HS-5 were cultured either alone or together at 1:5 (BMSC/MM) ratio and cell proliferation was measured using the CyQUANT™ direct cell proliferation assay.

### HR and NHEJ functional assays

For DNA repair assays MM cell lines (JJN3 and U266) carrying chromosomally integrated GFP-based HR or NHEJ reporter cassettes were used^27,28^. To evaluate HR and NHEJ efficiency in the absence of TRIM33, 10^6^ cells were first transfected with siRNA for TRIM33 and 24 h later co-transfected with 5 µg of an I-SceI-expressing plasmid together with 0.5 µg of pDsRed-N1 to normalize measurements with respect to the transfection efficiency. Live cells were selected by FSC/SSC gating, and live GFP + and DsRed + cells were quantified by flow cytometry. HR and NHEJ efficiency were calculated as the ratio of GFP + to DsRed + cells.

### Analysis of CoMMpass dataset

Publicly available patient data was accessed from the Multiple Myeloma Research Foundation (MMRF) (https://themmrf.org/) Relating Clinical Outcomes in MM to Personal Assessment of Genetic Profile (CoMMpass) dataset (IA15 release). This is a longitudinal observational study of over 1,000 newly diagnosed MM patients over a minimum period of 5 years. Data accessed and downloaded included patient demographics, clinical outcomes, structural variants (Whole Genome Sequencing), gene expression (RNA-Seq) and common genetic abnormalities assessed by fluorescence in-situ hybridization (FISH).

### Statistical analyses

All statistical analyses were performed using GraphPad Prism v8.0 software. The statistical test used is indicated in each figure legend and the following annotations used to represent statistical significance; *p < 0.05, **p < 0.01, ***p < 0.001, ****p < 0.0001.

## Results

### Low TRIM33 expression is associated with poor outcome

**Figure 1:**
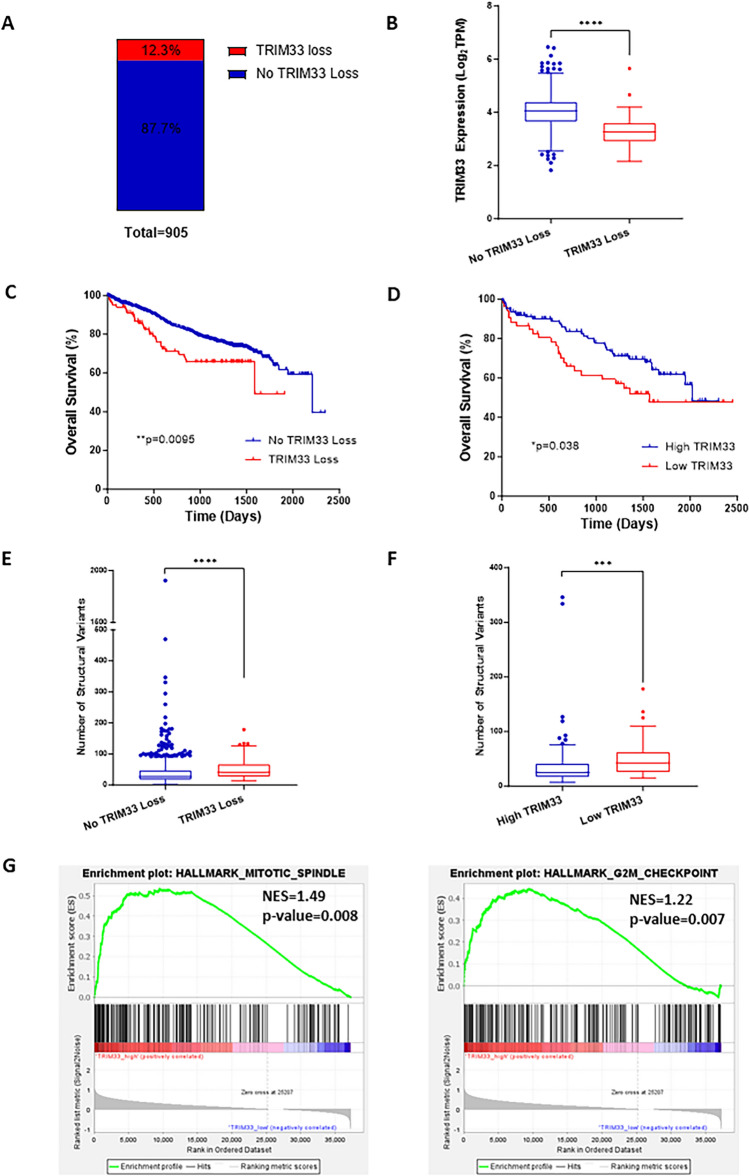
MM patients with loss/low expression of TRIM33 exhibit genomic instability. (**A**) 111 out of 905 (12.3%) patients from the CoMMpass dataset (IA15) exhibit copy number loss of TRIM33 (1p13.2). (**B**) TRIM33 expression in patients with no TRIM33 loss or TRIM33 loss; Mann Whitney U test ****p < 0.0001. (**C**) Kaplan–Meier analysis of overall survival (OS) of patients with TRIM33 CN loss or no loss; log-rank test, *p* = 0.0095 (**D**) Kaplan–Meier analysis of OS for patients with low TRIM33 expression (*n* = 53) or high TRIM33 expression (*n* = 126); Wilcoxon test, *p* = 0.03. (**E–F**) Number of structural variants (deletions, translocations, inversions, and duplications) for patients with TRIM33 loss and no TRIM33 loss (**E**) and for patients with low and high TRIM33 expression (**F**). Data analysed using the Mann Whitney U test, ****p < 0.0001, ***p < 0.001. (**G**) GSEA enrichment plots for patients with low vs high TRIM33 expression.

Analysis of the MMRF CoMMpass dataset identified that 12.3% of newly diagnosed MM patients had a copy number (CN) loss of TRIM33 (Fig. 1A) and demonstrated that patients with TRIM33 loss displayed significantly reduced TRIM33 gene expression (*p* < 0.0001; Fig. 1B). Further analysis revealed that TRIM33 loss correlated with significantly reduced overall survival (OS) compared to patients with no TRIM33 loss (median 1590 days vs. 2207 days, *p* = 0.0095; Fig. 1C). Patients with CN loss of TRIM33 often had interstitial deletions of 1p encompassing other genes in the region and as evident in Fig. 1B, there is also a proportion of patients without copy number loss of TRIM33 that exhibit low TRIM33 gene expression. We therefore analysed patients with low vs high TRIM33 expression and similarly observed reduced overall survival in patients with low TRIM33 expression (median 1564 days vs. 2018 days, *p* = 0.031, Fig. 1D). Decreased expression or deletion of TRIM33 has previously been linked with increased chromosome instability across a range of tumour types resulting from attenuation of both the spindle assembly checkpoint (SAC) and post-mitotic checkpoint^16^. Consistent with this, we show that patients with either TRIM33 loss or low TRIM33 gene expression display a significantly increased median number of structural variants (deletions, translocations, inversions, duplications) compared to those without loss (Fig. 1E; 41.5 vs 27; *p* < 0.0001) or with high TRIM33 expression (Fig. 1F; 42.5 vs 25; *p* = 0.0003), indicative of increased chromosomal instability. Furthermore, gene set enrichment analysis (GSEA) demonstrated significant negative enrichment of mitotic spindle and G2M checkpoint pathways (Fig. 1G) in patients with low TRIM33 expression suggesting there may be impairment of these pathways in the absence of TRIM33.

### TRIM33 is required for efficient DNA repair

**Figure 2:**
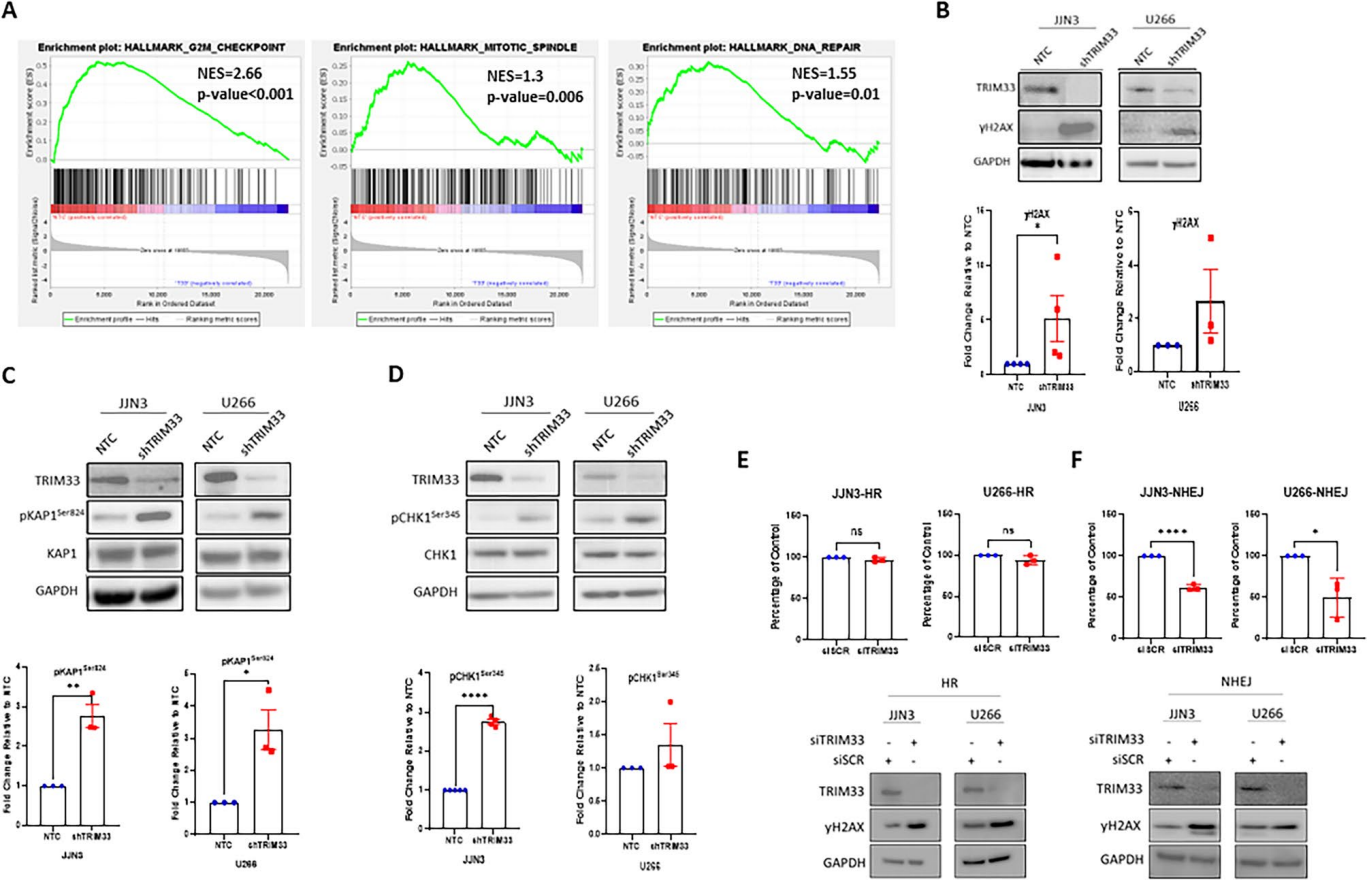
Loss of TRIM33 leads to endogenous DNA damage in MM cell lines. (**A**) GSEA enrichments plots for TRIM33 knockdown cells (shTRIM33) and non-targeting control (NTC) cells. (**B-D**) Representative Western blot analysis of (**A**) γH2AX, (**B**) pKAP1^Ser824^ and (**C**) pCHK1^Ser345^ in JJN3 and U266 NTC and shTRIM33 cells. Densitometric quantification was normalised to GAPDH (**B**) total KAP1 (**C**) or total CHK1 (**D**) and expressed as fold change compared to NTC. Data represent the mean ± SEM with *n* = 3 for all parts except JJN3 γH2AX and pCHK1^Ser345^ where *n* = 4. An unpaired t-test was used for statistical analysis; ****p < 0.0001, **p < 0.01, *p < 0.05. **(E)** Homologous recombination (HR) efficiency and **(F)** non-homologous end joining (NHEJ) efficiency in JJN3 and U266 cells transfected with siRNA against TRIM33 (siTRIM33), calculated as the ratio of GFP + to DsRed + cells and expressed as a percentage of scrambled control transfected cells (siSCR). Western blotting was performed to confirm knockdown of siRNA mediated knockdown of TRIM33 and increased endogenous DNA damage as measured using γH2AX. Data represent the mean ± SEM for 3 independent experiments; ****p < 0.0001, *p < 0.05.

To explore the effect of TRIM33 loss on MM cells in vitro, TRIM33 shRNA knockdown models were established using JJN3 and U266 cell lines (supplementary Fig. 2) and RNA-seq analysis was performed. Depletion of TRIM33 resulted in differential expression of 313 genes (fold change 2; *p* < 0.05; supplementary Table 1). In common with patient data, GSEA identified mitotic spindles and the G2M checkpoint pathways among the most negatively enriched pathways in TRIM33 knockdown cells (shTRIM33), along with DNA repair (Fig. 2A), aligning with previously reported roles for TRIM33 both in regulating mitotic checkpoints and as a regulator of DNA repair. To further explore the role of TRIM33 in DDR, we analysed steady-state expression of common DDR markers using Western blotting. Expression of γH2AX, a well-known marker for DNA double strand breaks (DSBs), was increased in shTRIM33 cells compared to cells transduced with a non-targeting control (NTC), suggesting an increase in DSB formation in the absence of TRIM33 (Fig. 2B). The kinases ATM and ATR become activated in response to DSBs to orchestrate the DDR. We assessed the levels of pKAP1^Ser824^, a downstream substrate of ATM and pCHK1^Ser345^ a downstream substrate of ATR, as markers of ATM and ATR activation respectively. An increase in phosphorylation of KAP1 (Fig. 2C) and CHK1 (Fig. 2D) was observed across both cell lines in the absence of TRIM33. DSB repair is mediated by two main pathways—homologous recombination (HR) and non-homologous end joining (NHEJ). To determine if the activity of either pathway was affected by loss of TRIM33, we used GFP-based HR and NHEJ fluorescent reporter assays. TRIM33 expression was silenced in JJN3 and U266 cells expressing a HR or NHEJ reporter plasmid using siRNA and repair efficiency was evaluated. While HR function was unaltered in the absence of TRIM33 (Fig. 2E), we observed a marked reduction in NHEJ-mediated repair (Fig. 2F), indicating that downregulation of TRIM33 in MM cells reduces NHEJ efficiency.

**Figure 3:**
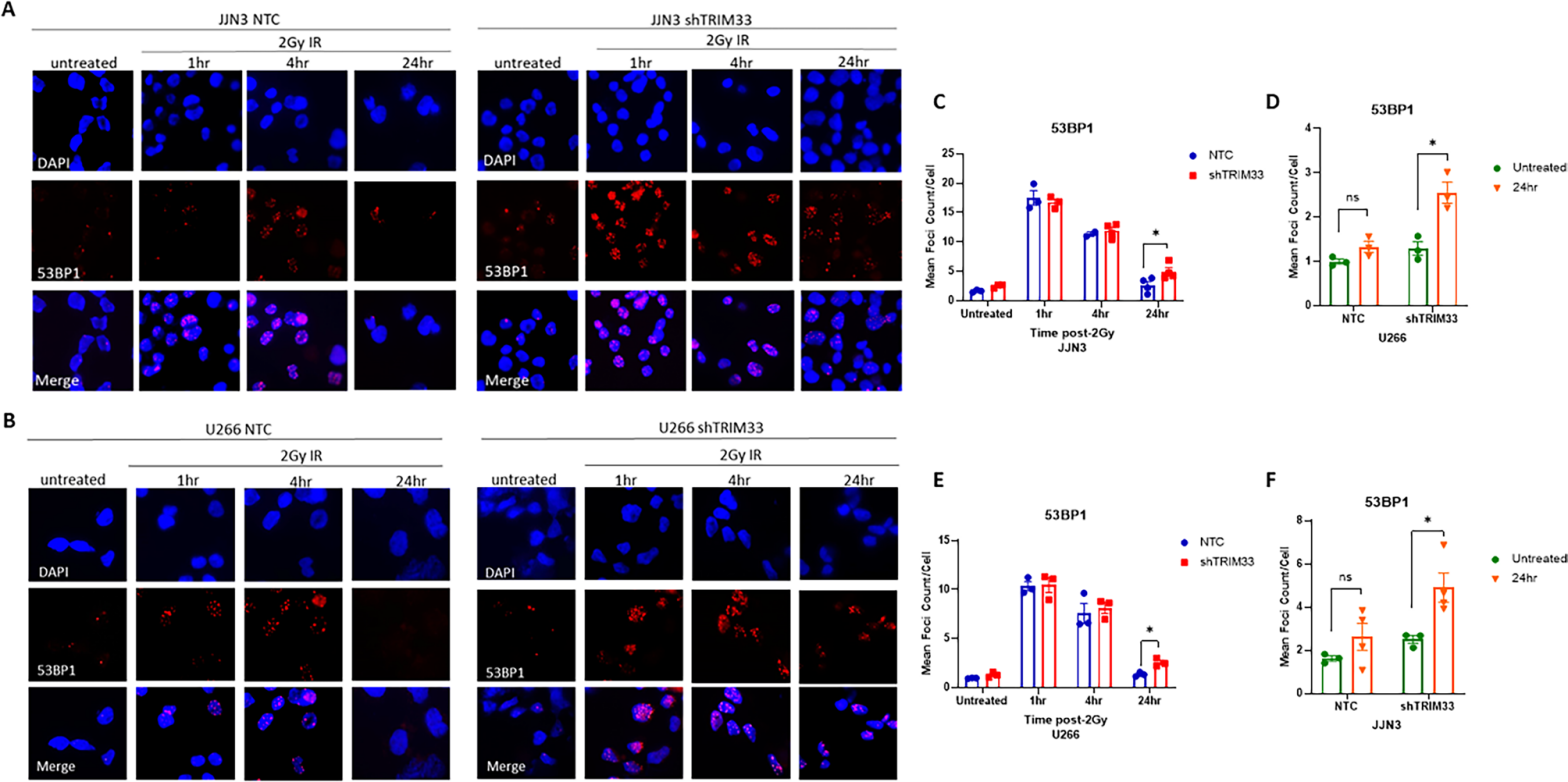
TRIM33 is required for efficient DSB repair. Representative images of JJN3 NTC and shTRIM33 cells (**A**) and U266 NTC and shTRIM33 cells (**B**) either untreated or following exposure to 2Gy IR at indicated time-points. Cells were stained with nuclear stain DAPI (blue) and 53BP1 (red). Images were captured at 60X magnification. (**C**) Mean number of 53BP1 foci per JJN3 NTC and shTRIM33 cells at indicated time-points following 2Gy IR. (**D**) Mean number of 53BP1 foci per JJN3 NTC and shTRIM33 cells when untreated or 24-h following 2Gy IR treatment. (**E**) Mean number of 53BP1 foci per U266 NTC and shTRIM33 cells at indicated time-points following 2Gy IR. (**F**) Mean number of 53BP1 foci per U266 NTC and shTRIM33 cells when untreated or 24-h following 2Gy IR treatment. 100 cells were examined per sample. Data represent the mean ± SEM with n = 3 and multiple t-tests used for statistical analysis, *p < 0.05.

As loss of TRIM33 resulted in an increase in spontaneous DSBs in the absence of an external stimulus, we investigated whether TRIM33 knockdown altered response to induced DSBs. NTC and shTRIM33 cells were exposed to 2Gy IR and the formation of 53BP1 foci was assessed over 24 h. As expected, the frequency of 53BP1 foci was markedly increased in all cell lines 1 h following IR (Fig. 3A–E). However, while control cells exhibit a time-dependent decrease in 53BP1 foci that returns to basal levels by 24 h following IR, shTRIM33 cells exhibit significantly higher 53BP1 foci levels compared to untreated shTRIM33 cells at 24 h (Fig. 3D and F) indicating a defective ability to repair DNA in a timely manner in the absence of TRIM33.

### PARP inhibition in combination with Bortezomib is a promising therapeutic option

**Figure 4:**
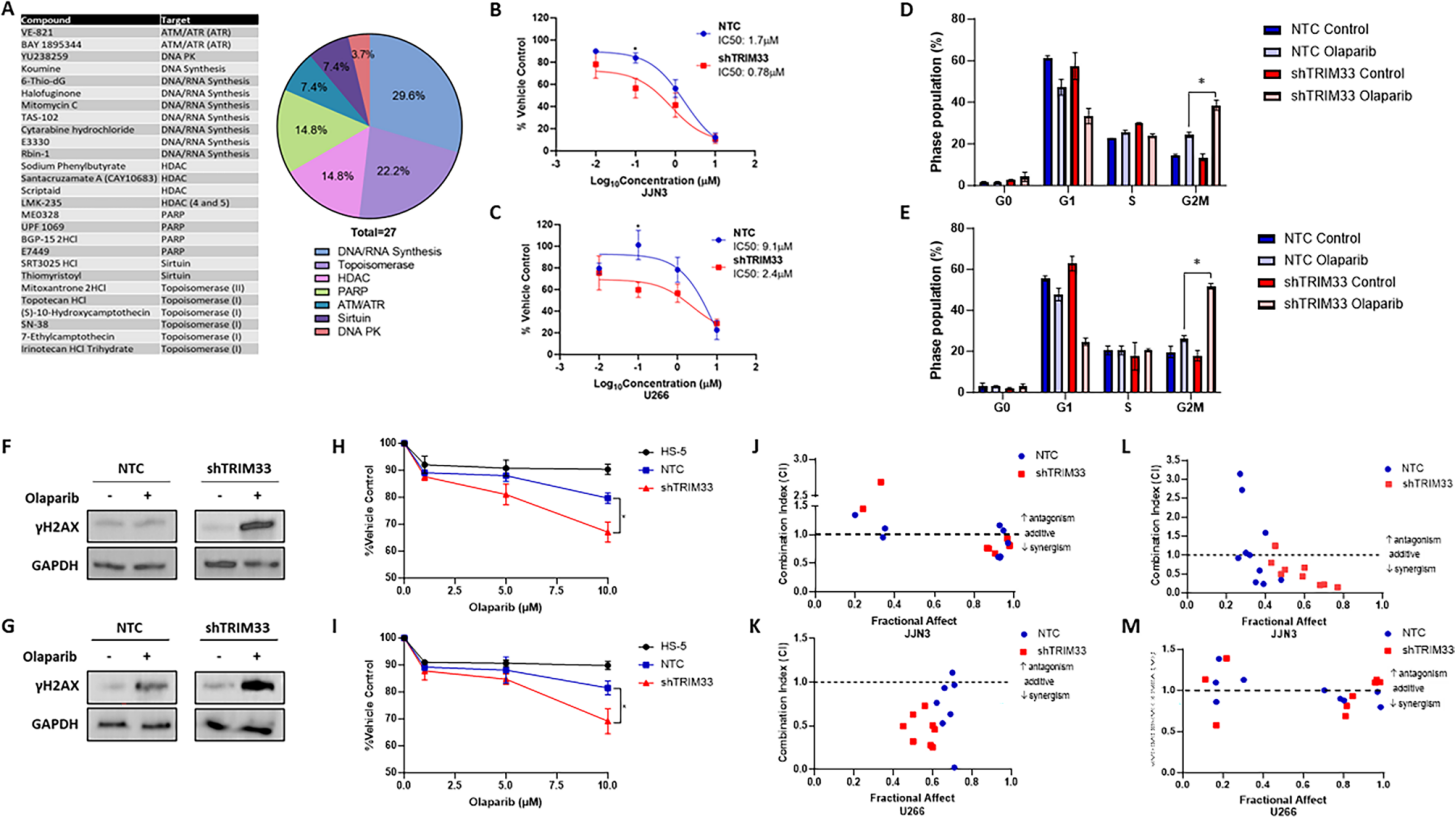
TRIM33 knockdown sensitizes cells to PARP inhibition. (**A**) The 27 unique compounds and targets displaying preferential cytotoxic activity in shTRIM33 cells and overview of the compound targets. (**B-C**) Olaparib dose response curves (10 nM–10 µM) in NTC and shTRIM33 JJN3 (**B**) and U266 (**C**) cells as assessed in clonogenic assays after 10 days. Data displayed as a percentage of vehicle control and represents the mean and SD of 3 independent experiments, *p < 0.05. (**D-E**) Cell cycle analysis of NTC and shTRIM33 JJN3 (**D**) and U266 (**E**) cells treated with 10 μM Olaparib for 48 h. Data displayed as percentage of cells in each cell cycle phase and represents the mean and SD of 3 independent experiments. (**F, G**) γH2AX expression in NTC and shTRIM33 JJN3 (**F**) and U266 (**G**) cells treated with 10 μM Olaparib for 48 h. (**H, I**) NTC and shTRIM33 JJN3 (**H**) and U266 (**I**) cells in co-culture with HS-5 bone marrow stromal cells (BMSCs) were treated with Olaparib (1, 5 or 10 μM) and viability assessed after 72 h. Data displayed as percentage of vehicle control and represents the mean and SD of 3 independent experiments, *p < 0.05. The effect of Olaparib on HS-5 cells alone is included for comparison. (**J, K**) NTC and shTRIM33 JJN3 (**J**) and U266 (**K**) cells were treated with pairwise combinations of Olaparib (1 µM, 5 µM and 10 µM) and bortezomib (2.5 nM, 5 nM, 10 nM). Combination indices were calculated using CompuSyn whereby > 1 indicates antagonism, 1 indicates an additive effect and < 1 indicates synergy. (**L, M**) NTC and shTRIM33 JJN3 (**L**) and U266 (**M**) cells in co-culture with HS-5 BMSCs were treated with pairwise combinations of Olaparib (1 µM, 5 µM and 10 µM) and bortezomib (2.5 nM, 5 nM, 10 nM) and combination indices calculated. Combination indices are presented as the mean of 3 independent experiments.

Given the evidence that TRIM33 functions within the DDR in MM cells, we explored whether loss of TRIM33 could sensitise cells to drugs that act on DNA damage/DDR pathways. Using a drug library comprised of 160 unique compounds, JJN3 NTC and JJN3 shTRIM33 cells were treated with 10 nM, 100 nM or 1 μM of each compound and cytotoxicity was assessed 24-, 48- and 72-h following treatment (supplementary Fig. 5). A full list of targets and methodology can be found in supplementary Table 2. In total, 27 compounds displayed preferential sensitivity in TRIM33 knockdown cells (Fig. 4A) and these were categorized into 7 different classes including those targeting DNA/RNA synthesis, topoisomerase inhibitors and PARP inhibitors. As TRIM33 is reported to function in PARP-dependent DDR, we focused on PARP inhibitors for further investigation.

NTC and shTRIM33 cells were treated with increasing doses of PARP inhibitor Olaparib and colony formation assessed following 10 days of treatment. In both JJN3 and U266 cell lines, shTRIM33 cells exhibited at least twofold increased sensitivity to Olaparib compared to control cells (Fig. 4B,C), characterised by an increased accumulation of cells in G2/M (Fig. 4D,E) and an increased accumulation of DNA damage (Fig. 4F,G). Furthermore, increased sensitivity of shTRIM33 cells was maintained when cells were co-cultured with the stromal cell line HS-5 (Fig. 4H,I). Proteasome inhibitors are a backbone of MM therapy, and we therefore investigated the combination of Olaparib and standard-of-care proteasome inhibitor bortezomib. Combination index (CI) analysis revealed synergism, indicated by a CI value of less than 1, between Olaparib and bortezomib, with a greater number of synergistic combinations observed in shTRIM33 cells (Fig. 4J,K). Synergistic effects were enhanced further when cells were co-cultured with bone marrow stromal cells (BMSCs) (Fig. 4J–M), with little effect observed on HS-5 cells alone (Supplementary Fig. 3), indicating that the combination of Olaparib and bortezomib can overcome the protective effect of BMSCs.

### TRIM33 regulates the stability of ALC1

**Figure 5:**
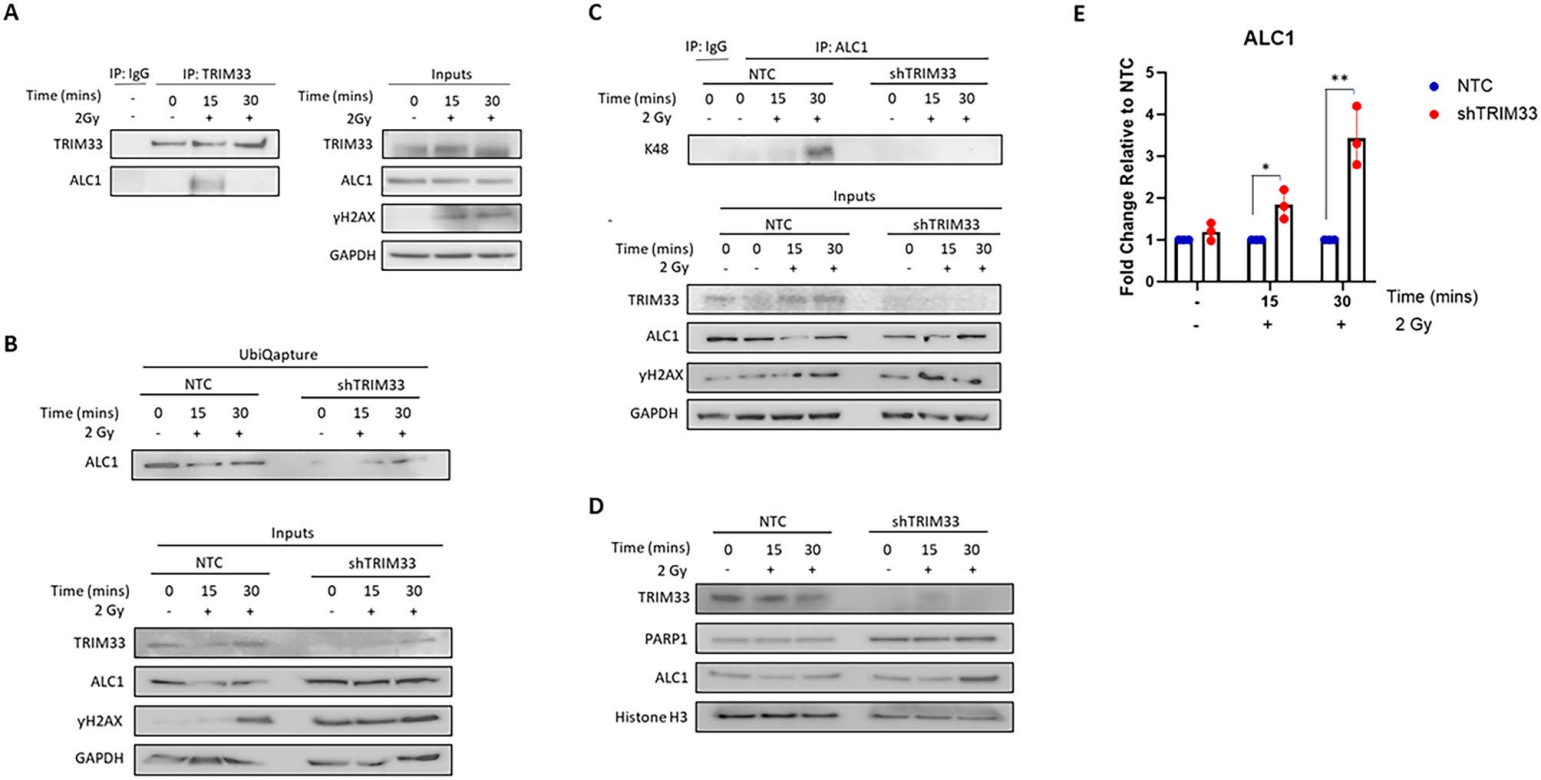
TRIM33 regulates ALC1 stability. (**A**) JJN3 cells were exposed to 2Gy IR for indicated time points and immunoprecipitation of TRIM33 and IgG isotype control performed and analysed by western blotting. Co-immunoprecipitation for ALC1 is shown on the left panel. Inputs are shown in the right panel demonstrating expression of TRIM33, ALC1 and DSB marker γH2AX in whole cell lysates. (**B**) JJN3 cells were exposed to 2Gy IR for indicated time points and immunoprecipitation of ubiquitinated proteins performed using UbiQapture and analysed by western blotting. Ubiquitinated ALC1 is shown in the top panel. Inputs are shown in the bottom panel demonstrating expression of TRIM33, ALC1 and γH2AX in whole cell lysates. (**C**) JJN3 cells were exposed to 2Gy IR for indicated time points and immunoprecipitation of K-48 ubiquitinated proteins and IgG isotype control was performed and analysed by western blotting. K-48 ubiquitinated ALC1 is shown in the left panel. Inputs are shown in the right panel demonstrating expression of TRIM33, ALC1 and γH2AX in whole cell lysates. Images are representative of one of three biological replicates (*n* = 3). (**D**) JJN3 cells were exposed to 2Gy IR for indicated time points, histones and histone-associated proteins were extracted and analysed by western blotting. Image is representative of one of three biological replicates (*n* = 3). (**E**) Densitometric quantification was normalised to GAPDH and expressed as fold change compared to NTC.

The chromatin remodeller ALC1 has recently been identified to regulate sensitivity to PARP inhibitors. As studies in HeLa cells demonstrated that TRIM33 functions in PARP-dependent DDR through interaction with and timely removal of ALC1 from sites of DNA damage, we wanted to explore this further. To determine if TRIM33 interacts with ALC1 in MM cells, co-immunoprecipitation was performed in JJN3 and U266 cells following exposure to 2Gy IR. While there was little interaction between TRIM33 and ALC1 in untreated cells, a strong interaction was observed 15 min after IR, which subsequently diminished by 30 min (Fig. 5A and Supplementary Fig. 4). Kulkarni and colleagues^17^ demonstrated that the RING domain of TRIM33 was required for timely dissociation of ALC1 from damaged DNA, suggesting that TRIM33 regulates ALC1 through ubiquitination. We demonstrate that in the absence of TRIM33, total levels of ALC1 ubiquitination are reduced (Fig. 5B) and we show that ALC1 is modified with K48-specific linkages following IR in control but not shTRIM33 cells (Fig. 5C). While control cells exhibit a reduction in ALC1 protein expression following irradiation, total and chromatin-bound ALC1 expression increases in shTRIM33 cells (Fig. 5D,E), indicating that TRIM33 promotes removal of ALC1 from damaged DNA through proteasomal degradation.

## Discussion

TRIM33 is a chromatin-associated RING E3 ligase that functions in many cellular processes including haematopoiesis, apoptosis, immunity and mitosis. Low expression or loss of TRIM33 is observed in several cancer types, and although its function is cell-type specific, TRIM33 is largely considered as a tumour suppressor. Del(1p) is a common abnormality in MM, associated with high-risk disease. Despite this, only a few genes have been identified to contribute to the adverse phenotype. TRIM33 is located on 1p13, a region often recurrently deleted in MM. In this study, we show that a subgroup of MM patients exhibit copy number loss and/or low expression of TRIM33, associated with poor overall survival and an increase in chromosomal structural variants. Pommier et al. previously reported that TRIM33 is required for adequate regulation of the spindle assembly checkpoint (SAC) which serves to regulate chromosome segregation and prevent progression from anaphase to metaphase within mitosis^16^. In the absence of TRIM33, SAC and post-mitotic checkpoints are attenuated enabling the accumulation of chromosomal abnormalities and increased tumour aggressiveness. Gene set enrichment analysis of MM patients with low TRIM33 expression and cell lines with TRIM33 knockdown corroborated these findings, revealing a negative enrichment of both mitotic spindle and G2M checkpoint pathways. Taken together this suggests that loss of TRIM33 in MM has a detrimental effect on mitosis leading to chromosomal instability, and this along with dysregulation of the G2/M checkpoint, which serves to prevent damaged cells from entering mitosis, could contribute to the accumulation of chromosomal aberrations we observe in patients.

In addition to mitotic defects, insufficient DDR can promote genomic instability and tumorigenesis. Previous studies have demonstrated that TRIM33 localises to sites of DNA damage and is involved in the PARP-dependent DDR. Here we show that TRIM33 knockdown cells are negatively enriched for DNA repair pathways suggesting a DDR defect. Knockdown of TRIM33 in MM cell lines resulted in spontaneous accumulation of DSB marker γH2AX, along with activation of DNA damage sensing kinases ATM and ATR. Moreover, in the absence of TRIM33, MM cells failed to repair induced DSBs in a timely manner with DSBs persisting 24 h following IR, indicating a defect in DSB repair. If not efficiently repaired, DSBs can promote genomic instability and tumorigenesis. To counteract this, cells have evolved two main DSB repair pathways—HR and NHEJ. Using reporter assays we show that TRIM33 loss greatly reduced NHEJ efficiency, with no effect observed on HR efficiency. This differed from previous work showing a reduction in HR in the absence of TRIM33, again highlighting it’s cell type specific function^19^.

The observed DDR deficiency in TRIM33 knockdown MM cells, along with previous reports of enhanced sensitivity to DNA damaging agents in the absence of TRIM33, led us to investigate therapeutic vulnerabilities in our cell line models. High-throughput screening of DNA damaging compounds identified 27 compounds across a range of drug classes that displayed increased efficacy in TRIM33 knockdown cells compared to controls. Intriguingly, PARP inhibitors exhibited enhanced sensitivity across both TRIM33 knockdown models. PARP inhibitors are widely accepted to demonstrate synthetic lethality in HR-deficient, but not NHEJ-deficient, tumours by inducing an accumulation of single strand breaks; these are subsequently converted to DSBs that cannot be repaired by HR due to an underlying HR deficiency^22^. To explore the mechanism behind the increased sensitivity in TRIM33 knockdown cells, which demonstrated an NHEJ-deficiency, we investigated the interaction of TRIM33 with ALC1. Recent studies have identified ALC1 as a regulator of PARP inhibitor response^23–25^. Loss of ALC1 was demonstrated to potentiate sensitivity through enhanced PARP2 trapping at DNA breaks^25^. While another study found that the absence of ALC1 resulted in post-replicative DNA gaps and a dependence on HR for repair^23^. As highlighted previously, TRIM33 is required for timely removal of ALC1 from sites of DNA damage^17^. In line with previous studies, we demonstrate a rapid interaction between TRIM33 and ALC1 in our cell line models. Furthermore, we show for the first time that loss of TRIM33 abrogates K48 linked ubiquitination of ALC1, leading to retention of ALC1 on chromatin following DNA damage. We hypothesise that prolonged retention of ALC1 in the absence of TRIM33 leads to dysregulated ALC1 activity at DNA damage sites resulting in a similar phenomenon to loss of ALC1. PARP inhibitors also demonstrated synergistic activity with standard of care therapy bortezomib in our model cell lines, particularly in TRIM33 knockdown cells. Bortezomib impairs HR through depletion of nuclear ubiquitin which abrogates H2AX polyubiquitination and subsequent recruitment of essential DDR components^26^. This has previously been demonstrated to result in enhanced sensitivity to PARP inhibitors in MM. Here we show that TRIM33 knockdown further heightens sensitivity to the combination of bortezomib and PARP inhibition.

## Conclusion

**Figure 6:**
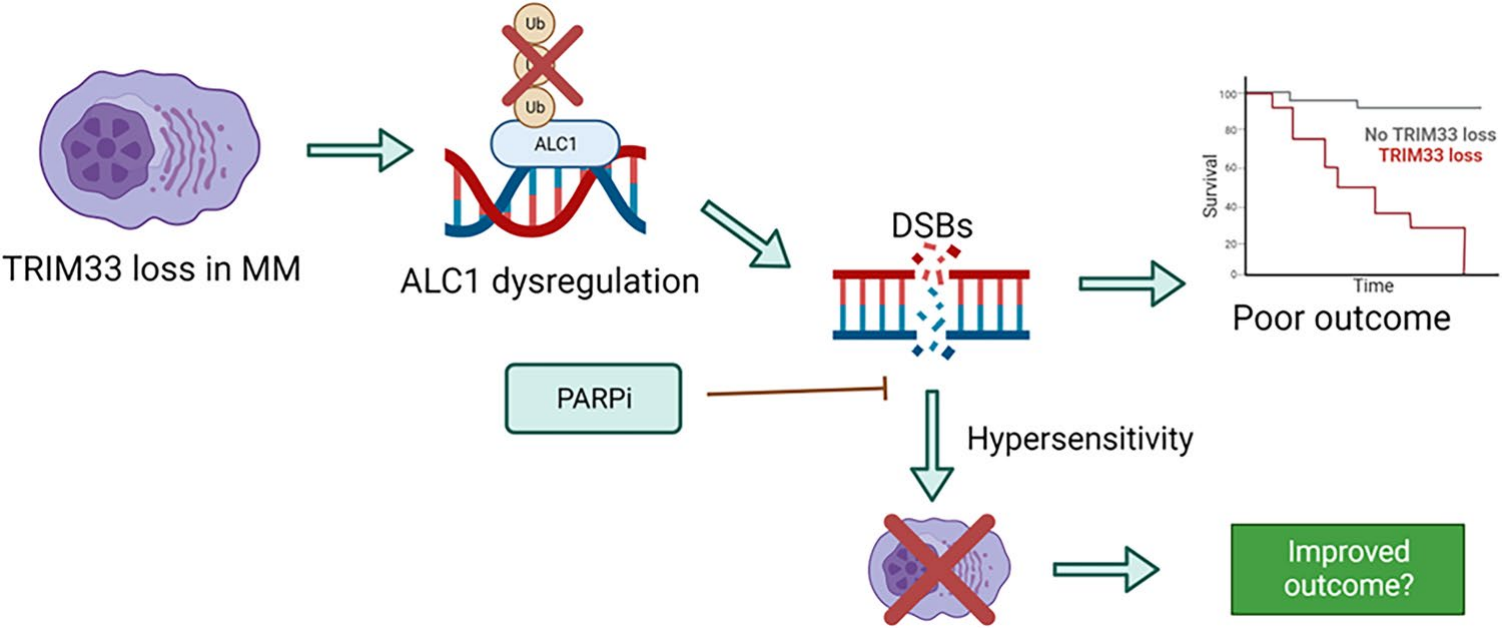
Graphical summary. TRIM33 loss in MM cells disrupts ubiquitination of ALC1 at DSB sites, leading to defective DSB repair, increased genomic instability and poor patient outcome; this results in sensitivity to PARP inhibition.

In summary, this study shows that loss or low expression of TRIM33 in MM is associated with high-risk disease characterized by chromosomal abnormalities and defective DDR. We show that loss of TRIM33 results in sensitivity to PARP inhibition and hypothesise that this is driven by dysregulation of ALC1 activity at sites of DNA damage (Fig. 6). We demonstrate that co-treatment with bortezomib and PARP inhibitors leads to enhanced cytotoxicity in the absence of TRIM33, suggesting this combination could provide a therapeutic option for a subset of MM patients with loss of TRIM33.

### Supplementary Information

Supplementary Information 1.

Supplementary Information 2.

## Data Availability

All data generated as part of this study are included in the article or will be made available on request to lisa.crawford@qub.ac.uk.

## Abbreviations

ALC1: Amplified in liver cancer 1
BMSC: Cone marrow stromal cell
CN: Copy number
DDR: DNA damage response
DSB: Double strand break
GSEA: Gene set enrichment analysis
Gy: Gray
HR: Homologous recombination
IR: Infrared radiation
MM: Multiple myeloma
NHEJ: Non-homologous end joining
NTC: Non-targeting control
OS: Overall survival
shTRIM33: ShRNA targeting TRIM33
TRIM33: Tripartite motif containing 33
