# Supplementary material for: TRIM33 loss in multiple myeloma is associated with genomic instability and sensitivity to PARP inhibitors
Source: Sci Rep. 2024 Apr 16;14:8797. doi: 10.1038/s41598-024-58828-8 (PMC11021562; doi:10.1038/s41598-024-58828-8)

Full Length Westerns

Below are full length Western blots related to images presented in the main manuscript and supplementary figures. Where possible we present full length membranes, however, there are some instances where membranes were cut prior to hybridization and/or the ladder and membrane edge have not saved as part of the image.

Figure 2

2B Left panel (JJN3)

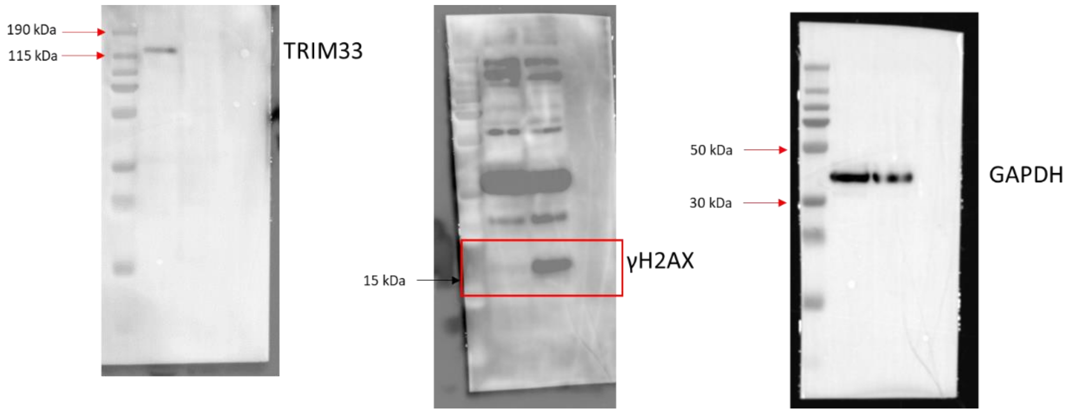

2B Right panel (U266)

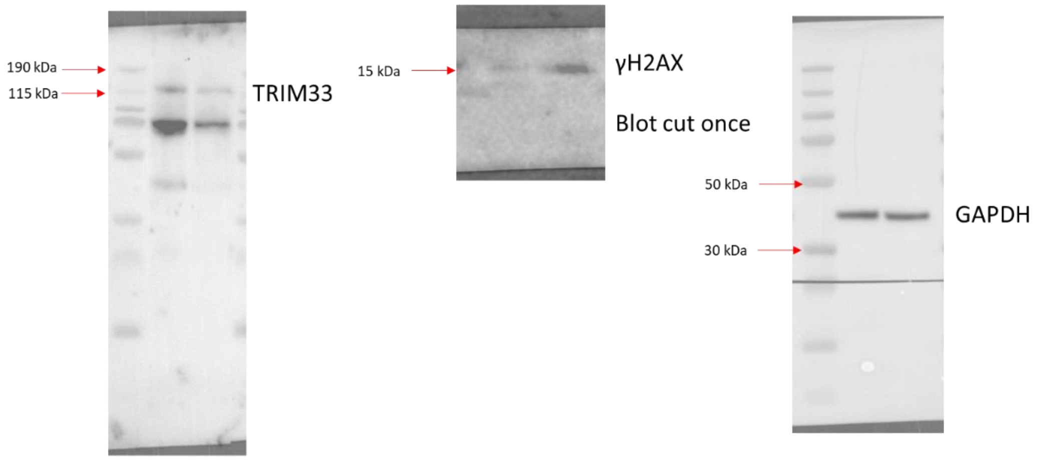

2C Left panel (JJN3)

pKAP1

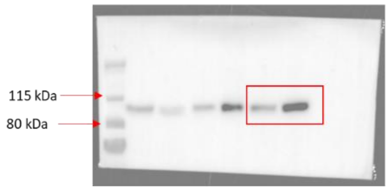

KAP1

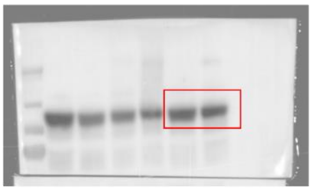

GAPDH

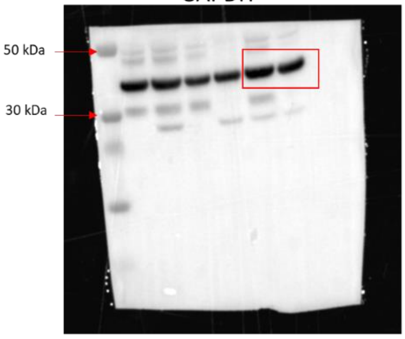

TRIM33

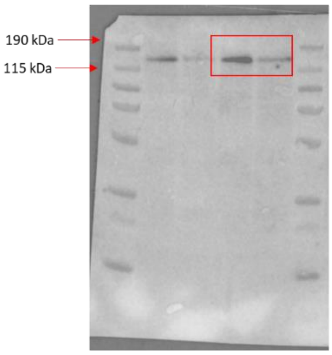

GAPDH

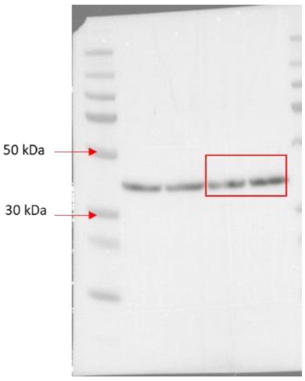

2C Right panel (U266)

pKAP1

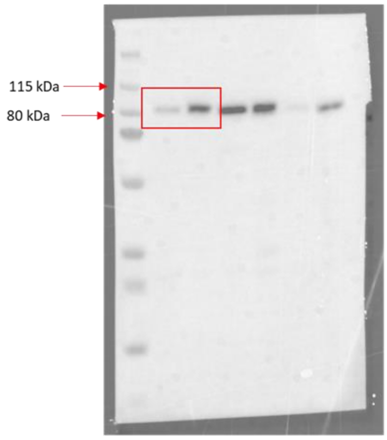

KAP1

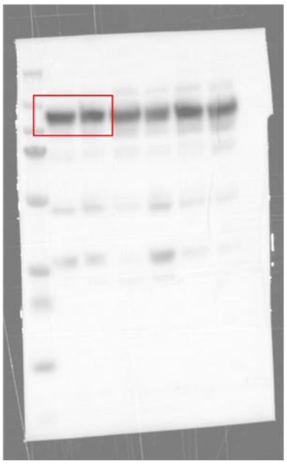

GAPDH

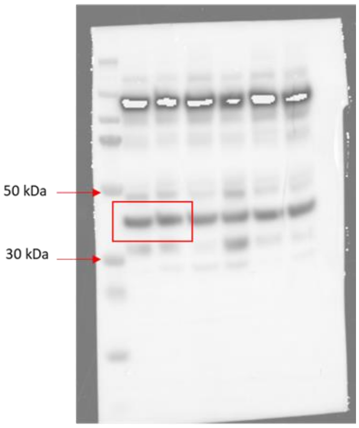

TRIM33

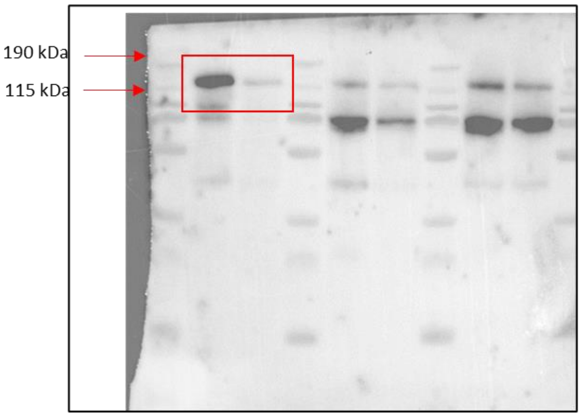

2D left panel (JJN3)

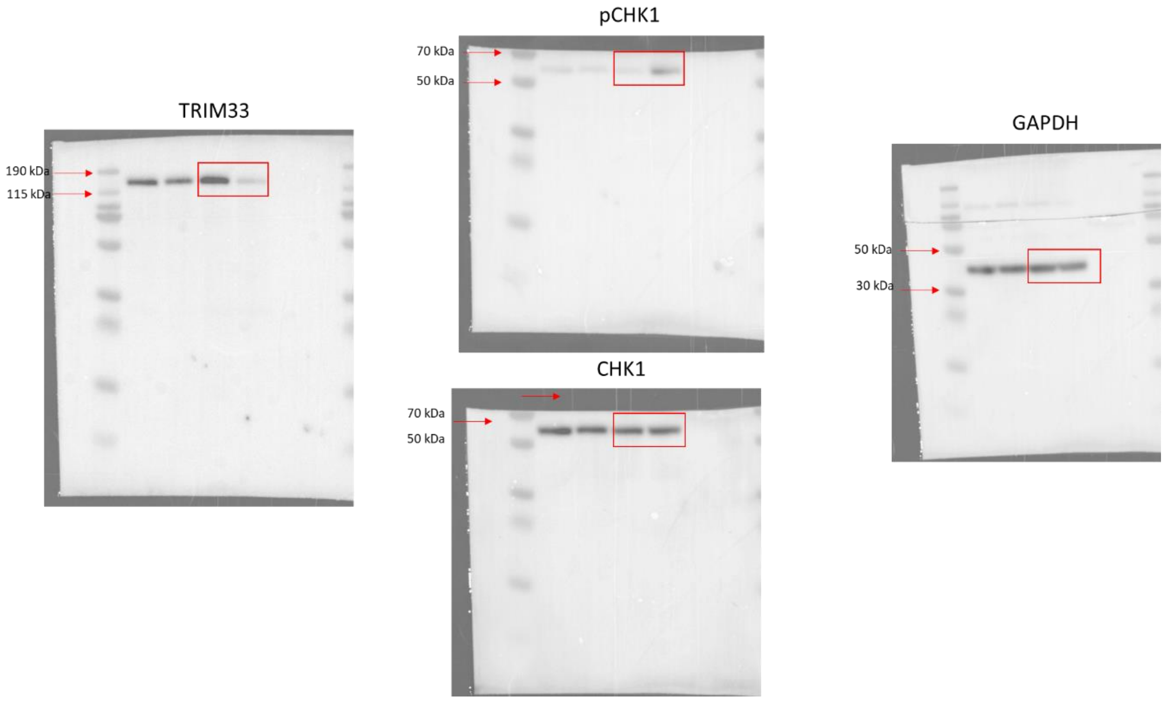

2D right panel (U266)

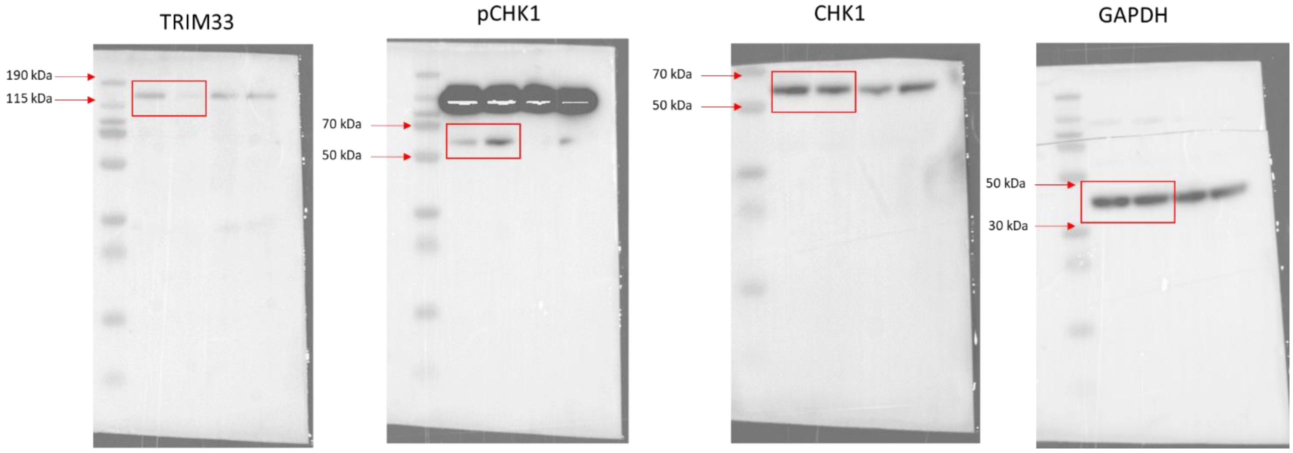

2E HR blots

Right panel (JJN3)

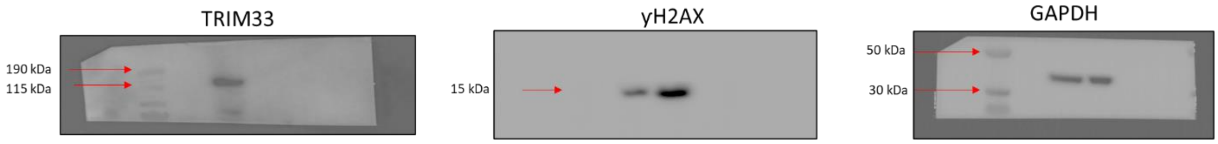

Left panel (U266)

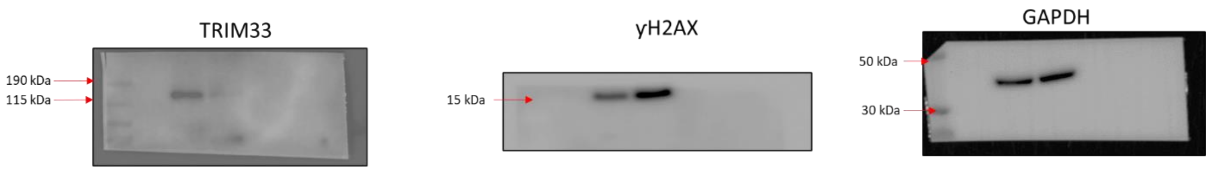

2F NHEJ blots

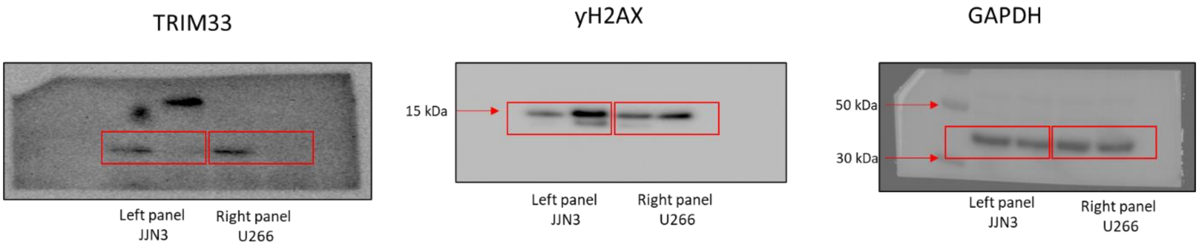

Figure 4

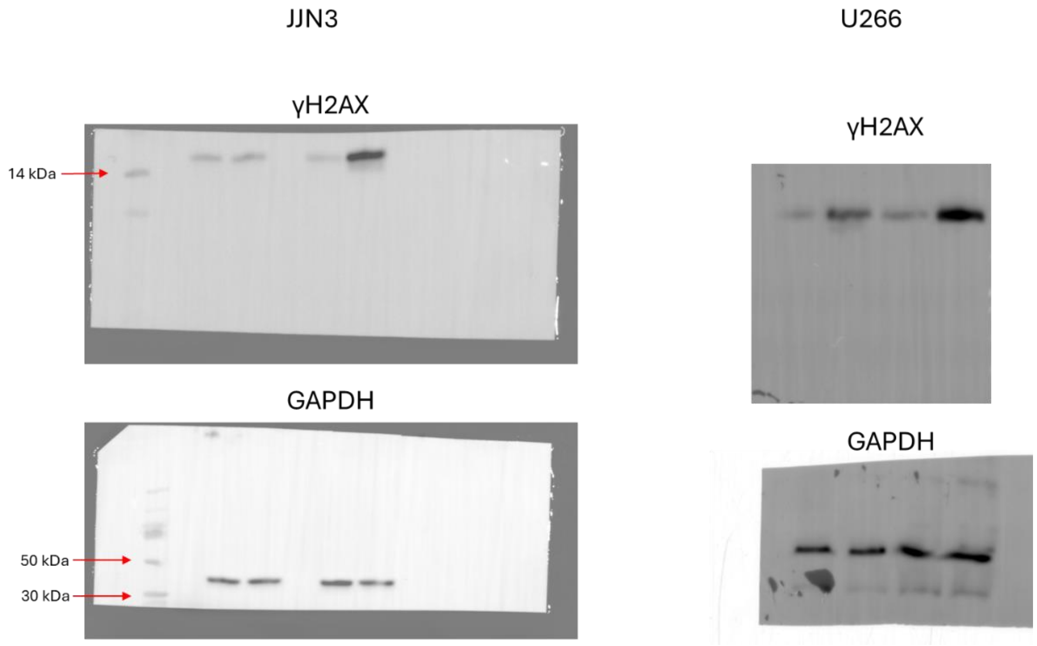

Figure 5

5A

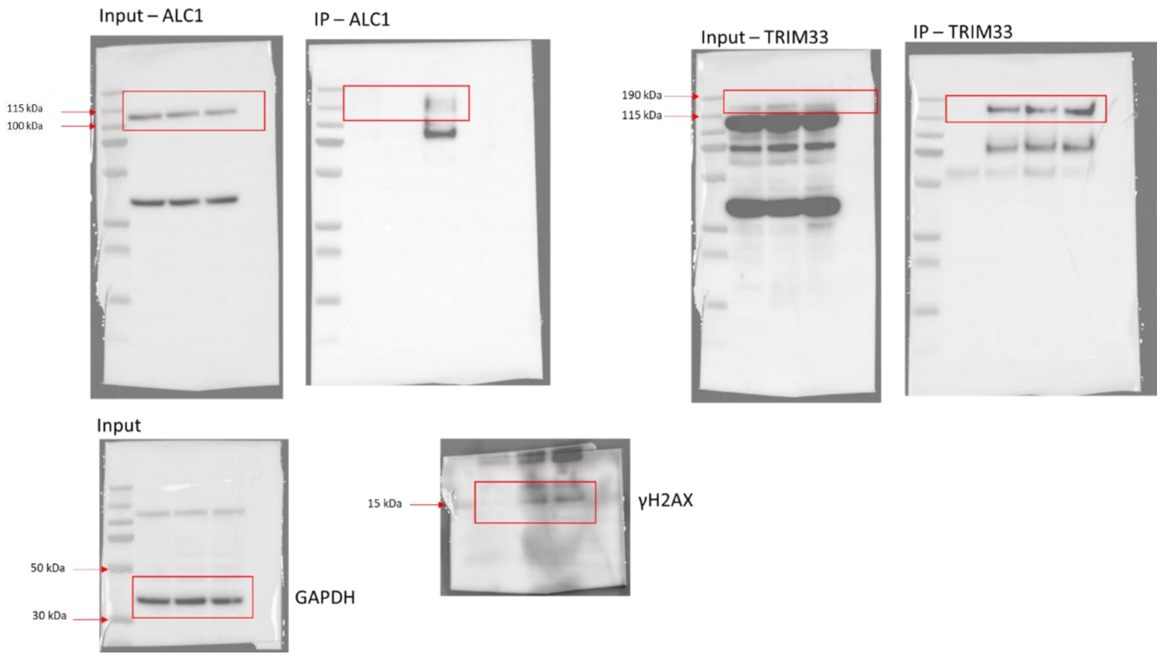

5B

ALC1

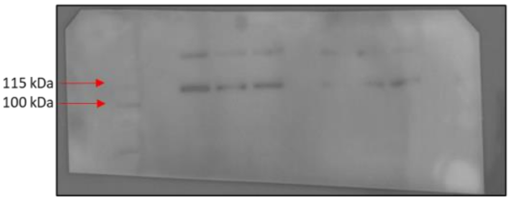

INPUT

TRIM33

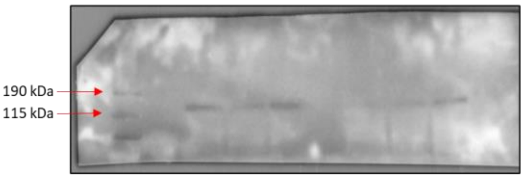

γH2AX

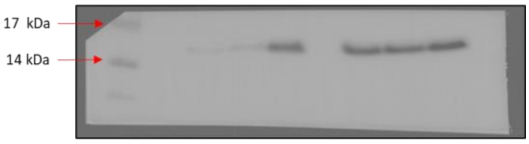

ALC1

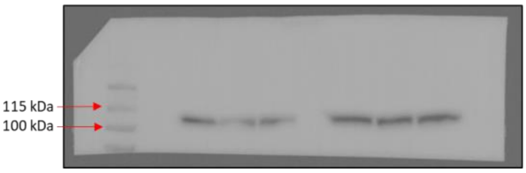

GAPDH

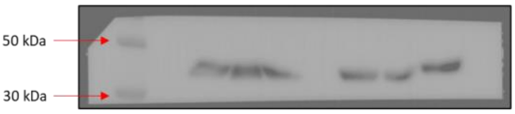

5C

TRIM33

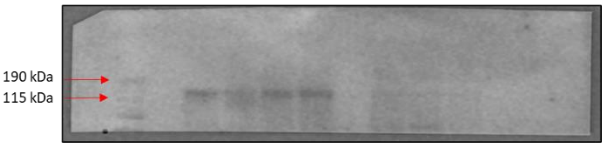

K48 Linkages

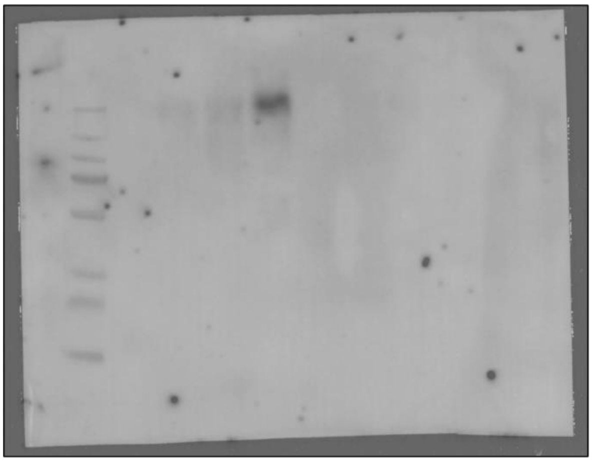

ALC1

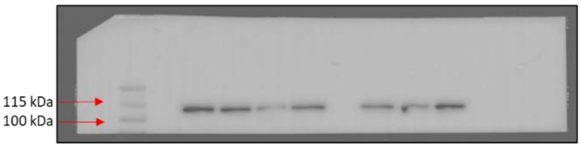

γH2AX

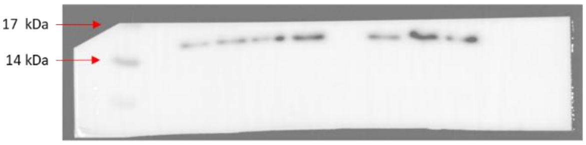

GAPDH

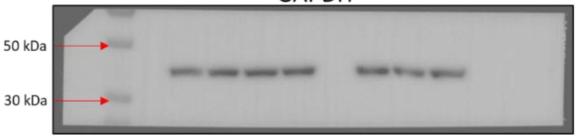

5D

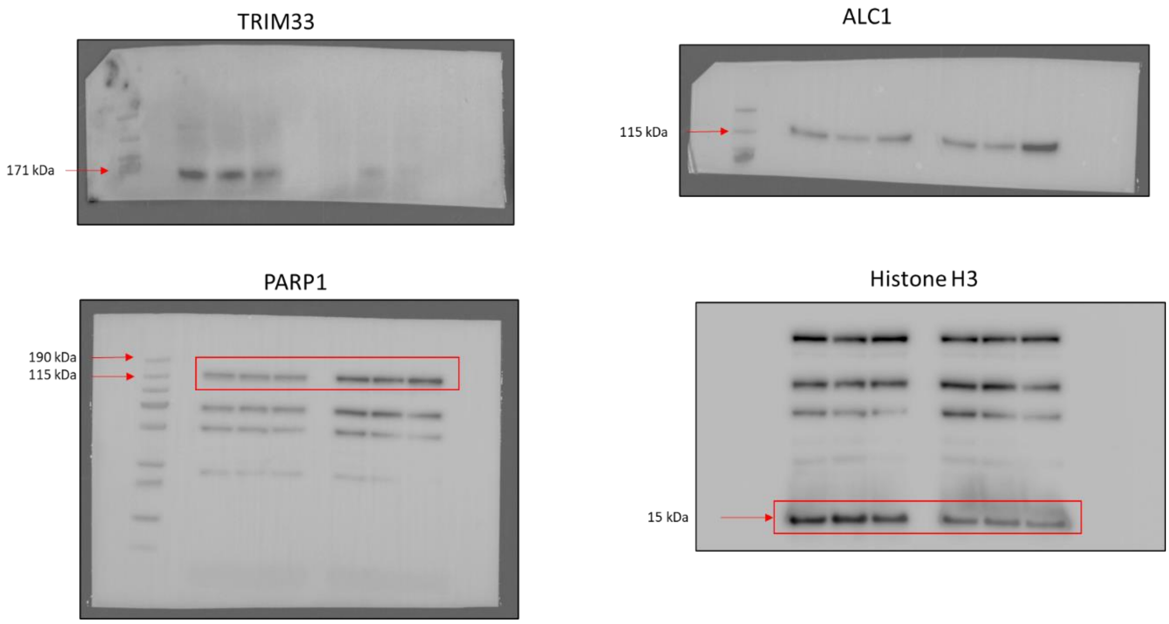
TRIM33
171 kDa
ALC1
115 kDa
PARP1
190 kDa
115 kDa
Histone H3
15 kDa

Supplementary Figure 1

Supplementary Figure 1A

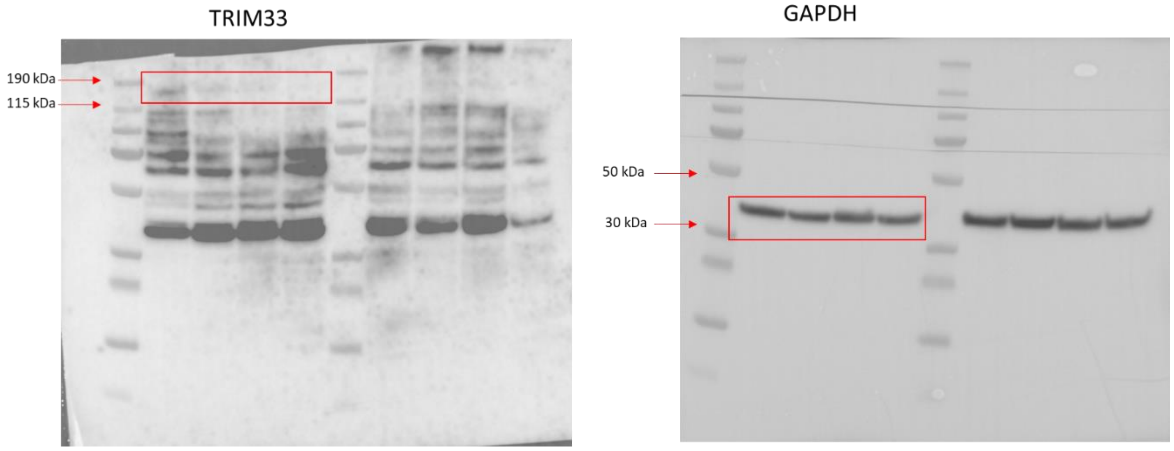

Supplementary Figure 1B

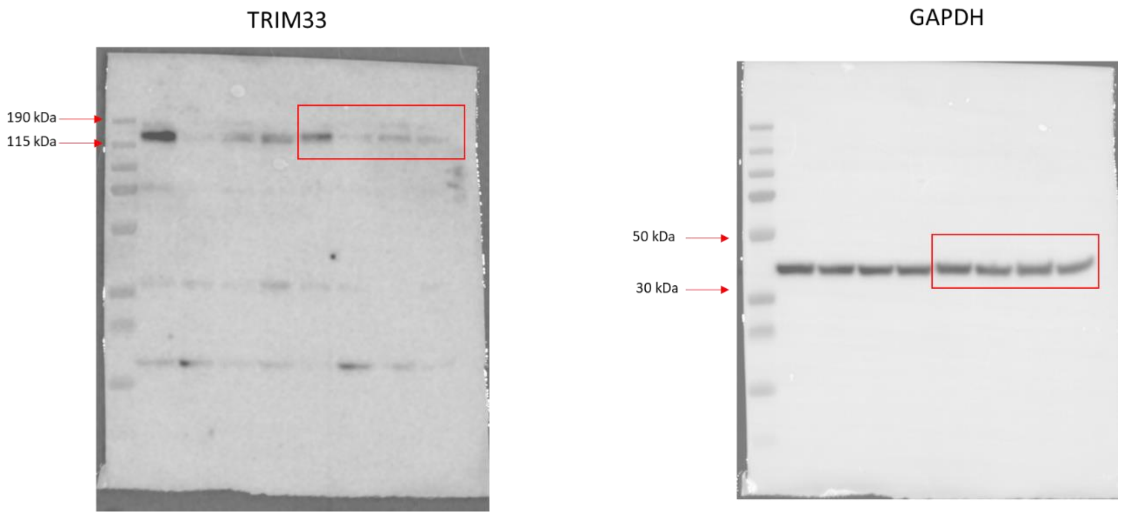

Supplementary Figure 3

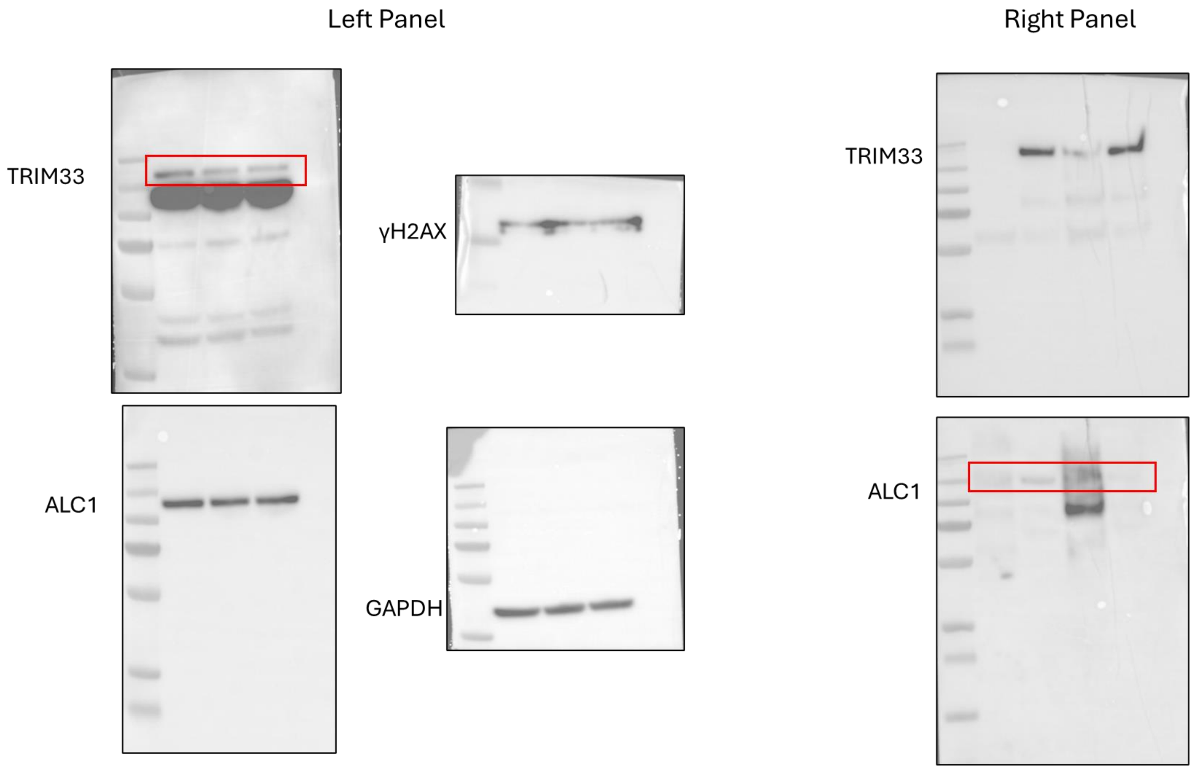

Supplement: Supplementary file 1 — Supplementary Information 1. [file 41598_2024_58828_MOESM1_ESM.pdf]
