# Supplementary material for: TRIM33 loss in multiple myeloma is associated with genomic instability and sensitivity to PARP inhibitors
Source: Sci Rep. 2024 Apr 16;14:8797. doi: 10.1038/s41598-024-58828-8 (PMC11021562; doi:10.1038/s41598-024-58828-8)
Supplement: Supplementary file 2 — Supplementary Information 2. [file 41598_2024_58828_MOESM2_ESM.docx]

**Supplementary Figure Legends**

**Supplementary Figure 1.** shRNA knockdown of TRIM33 in MM cell lines

MM cell lines **(A)** JJN3 and **(B)** U266, were transduced with a stable NTC or one of three shRNAs against TRIM33. Western blot analysis for TRIM33 was performed and densitometry analysis of TRIM33 relative to GAPDH and normalized to NTC. Data represent the mean ± SEM of at least 3 independent experiments. One-way ANOVA was used for statistical analysis; ****p<0.0001 **p<0.01. Cells transduced with shRNA#3 were used for all experiments.

.

**Supplementary Figure 2.** Olaparib and bortezomib combinations have little effect on HS-5 bone marrow stromal cells. HS-5 cells were treated with combinations of Olaparib (Olap) and bortezomib (Btz) as indicated. Data displayed as a percentage of vehicle control and represents the mean and SD of 3 independent experiments.

**Supplementary Figure 3.** TRIM33 and ALC1 interact in response to IR in U266 cell line.

U266 cells were exposed to 2Gy IR for indicated time points and immunoprecipitation of TRIM33 and IgG isotype control performed and analysed by western blotting. Co-immunoprecipitation for ALC1 is shown on the left panel. Inputs are shown in the right panel demonstrating expression of TRIM33, ALC1 and DSB marker γH2AX in whole cell lysates.

**Supplementary Figure 4**

**(A)** Schematic overview of DNA damage/DNA repair compound screening. JJN3 NTC and shTRIM33 cells were treated with 160 unique compounds at 100 nM and 1µM concentrations, and cytotoxicity assessed over 72 hours using CellTox Green™ cytotoxicity assay. Compounds meeting selection criteria when screened at **(B)** 100 nM and **(C)** 1 μM doses. Left panel – Normalized cytotoxicity in NTC and shTRIM33 cells meeting criteria. The dotted line indicates 50% cytotoxicity. Right panel – the cytotoxicity fold change in shTRIM33 cells relative to NTC cells in response to indicated compounds. Time-points of cytotoxicity assessment are indicated above. Dotted line indicates 1.5-fold change.

**
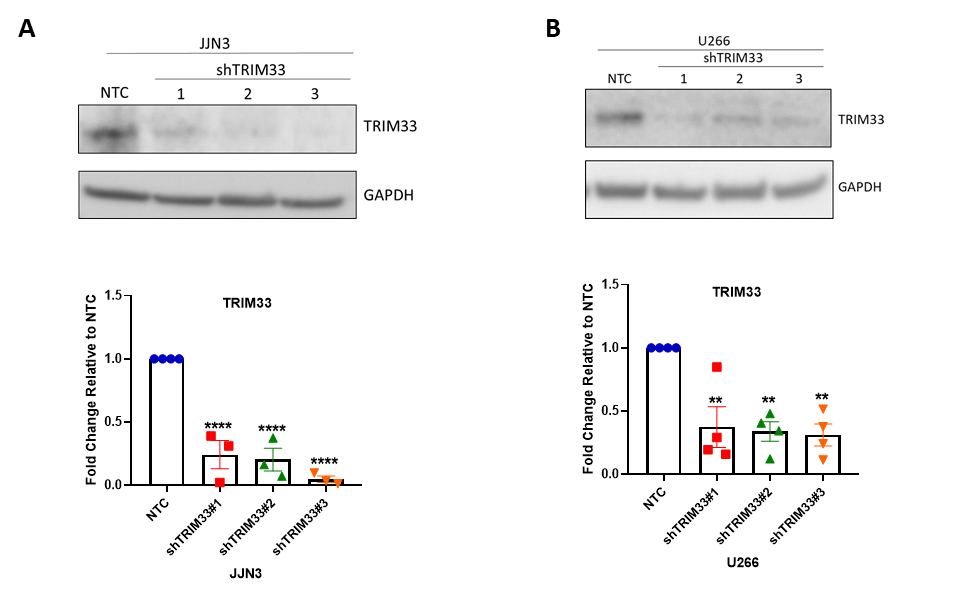
Supplementary Figure 1**

**Supplementary Figure 2**


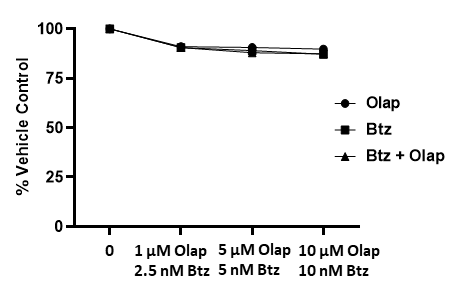


**Supplementary Figure 3**

**
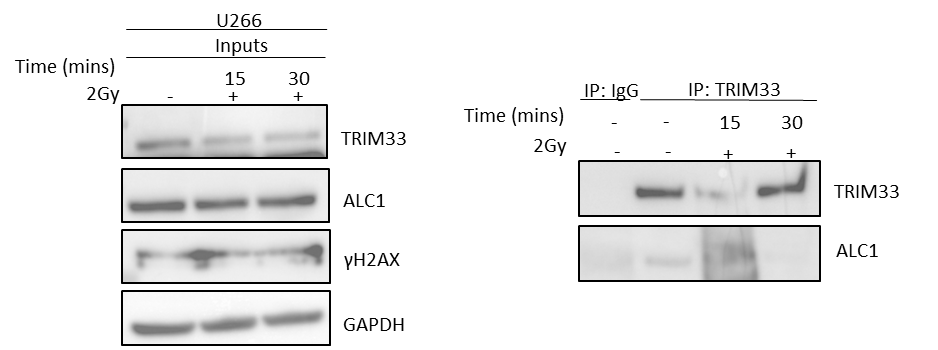
**

**
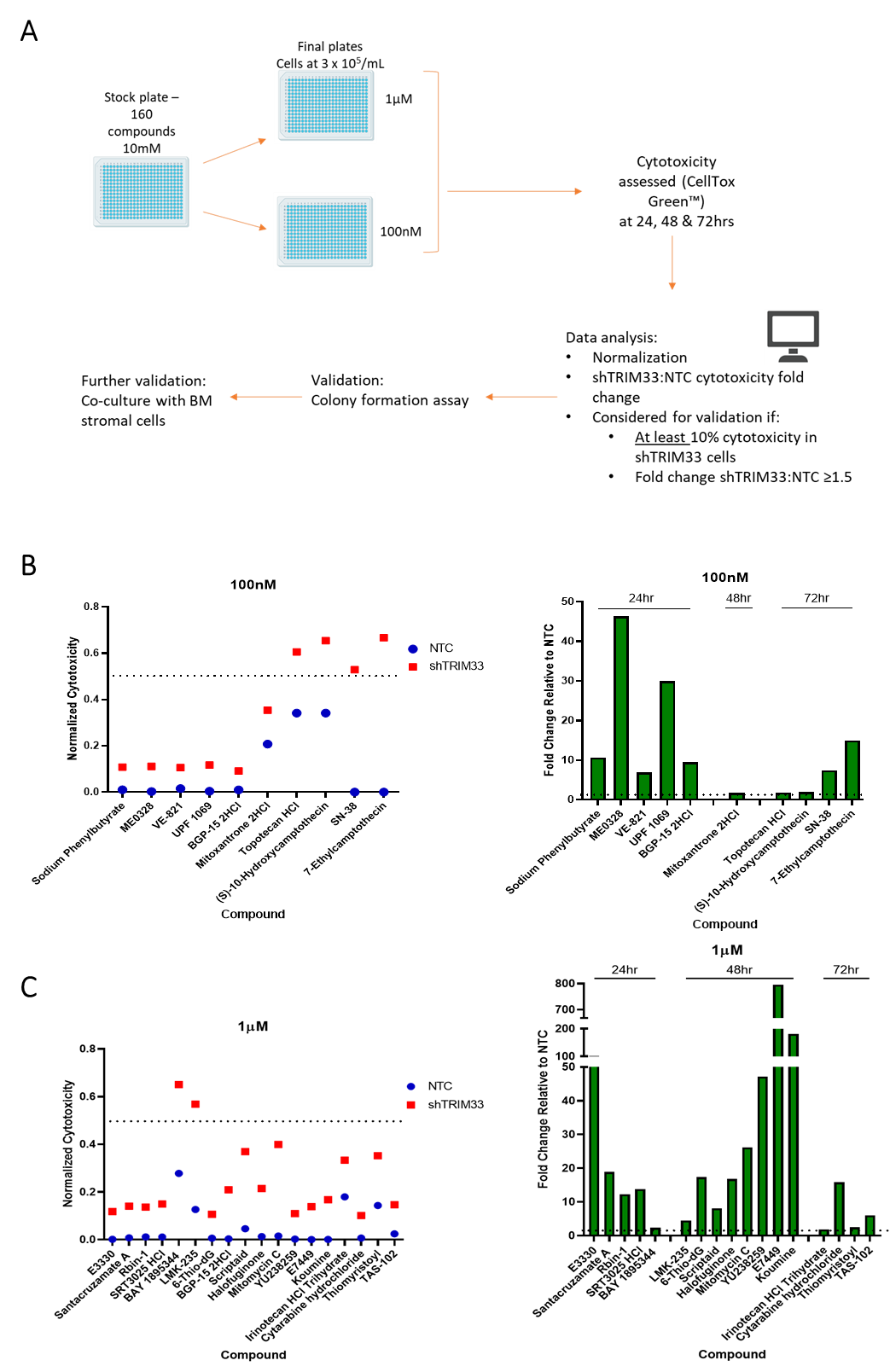
Supplementary Figure 4**

**Supplementary Tables**

**Supplementary Table 1.** Differentially expressed genes upon TRIM33 depletion

| Ensembl gene ID | Gene name | Average expression shTRIM33 | Average expression NTC | log2FoldChange | p.value | adj.p.value |
| --- | --- | --- | --- | --- | --- | --- |
| ENSG00000140563 | MCTP2 | 5.399607206 | 194.3612009 | -5.155401473 | 1.03E-29 | 1.89E-25 |
| ENSG00000189060 | H1F0 | 13055.59364 | 2769.290139 | 2.236954863 | 3.74E-28 | 3.42E-24 |
| ENSG00000111335 | OAS2 | 899.992104 | 157.9100833 | 2.508879538 | 5.14E-24 | 2.71E-20 |
| ENSG00000172927 | MYEOV | 310.4933975 | 26.78109741 | 3.524846337 | 5.94E-24 | 2.71E-20 |
| ENSG00000165023 | DIRAS2 | 73.01381427 | 451.1129427 | -2.628586927 | 1.89E-22 | 6.91E-19 |
| ENSG00000260916 | CCPG1 | 912.4011982 | 186.1676486 | 2.288729354 | 3.53E-19 | 1.00E-15 |
| ENSG00000130707 | ASS1 | 267.7239131 | 26.55319579 | 3.319428341 | 3.83E-19 | 1.00E-15 |
| ENSG00000172578 | KLHL6 | 422.3640931 | 77.40616513 | 2.443732504 | 6.06E-19 | 1.38E-15 |
| ENSG00000263006 | ROCK1P1 | 638.6050966 | 72.5354748 | 3.13090876 | 8.96E-19 | 1.64E-15 |
| ENSG00000101187 | SLCO4A1 | 151.0072532 | 765.3154216 | -2.34496472 | 2.01E-18 | 3.34E-15 |
| ENSG00000104413 | ESRP1 | 156.5836806 | 15.62970614 | 3.293460028 | 2.08E-17 | 2.53E-14 |
| ENSG00000130487 | KLHDC7B | 415.533112 | 46.3660395 | 3.147955087 | 7.22E-17 | 8.24E-14 |
| ENSG00000149294 | NCAM1 | 39.67885833 | 272.3745988 | -2.762721913 | 9.52E-17 | 1.02E-13 |
| ENSG00000163618 | CADPS | 11.50440825 | 110.2792816 | -3.277767351 | 1.18E-16 | 1.19E-13 |
| ENSG00000100889 | PCK2 | 4261.571601 | 743.6469214 | 2.517659698 | 1.46E-16 | 1.41E-13 |
| ENSG00000144481 | TRPM8 | 1151.626843 | 171.1228336 | 2.74682188 | 5.26E-16 | 4.81E-13 |
| ENSG00000172216 | CEBPB | 2098.827518 | 494.6546394 | 2.083204166 | 1.06E-15 | 9.21E-13 |
| ENSG00000115107 | STEAP3 | 36.83023243 | 201.1067938 | -2.455227951 | 1.37E-15 | 1.14E-12 |
| ENSG00000121895 | TMEM156 | 170.3545232 | 28.3028512 | 2.57714382 | 1.98E-15 | 1.51E-12 |
| ENSG00000167613 | LAIR1 | 281.5660734 | 58.05832111 | 2.288523209 | 8.20E-15 | 5.17E-12 |
| ENSG00000019582 | CD74 | 3531.325111 | 880.2792884 | 2.003713724 | 2.50E-14 | 1.47E-11 |
| ENSG00000101255 | TRIB3 | 6982.466116 | 1699.758465 | 2.038334496 | 3.39E-14 | 1.93E-11 |
| ENSG00000277449 | CEBPB-AS1 | 209.2236866 | 47.35004747 | 2.135747503 | 6.21E-14 | 3.34E-11 |
| ENSG00000104970 | KIR3DX1 | 87.93037817 | 6.141260619 | 3.879869284 | 6.17E-14 | 3.34E-11 |
| ENSG00000119242 | CCDC92 | 353.6107872 | 86.61142251 | 2.023656913 | 2.50E-13 | 1.21E-10 |
| ENSG00000137821 | LRRC49 | 687.1211931 | 155.4991724 | 2.138479551 | 2.52E-13 | 1.21E-10 |
| ENSG00000253616 | AC107959.3 | 265.2556728 | 42.24377385 | 2.632976597 | 3.02E-13 | 1.41E-10 |
| ENSG00000011028 | MRC2 | 70.17348515 | 377.0676027 | -2.434615263 | 3.44E-13 | 1.57E-10 |
| ENSG00000018625 | ATP1A2 | 126.5597761 | 11.04116287 | 3.47243868 | 4.69E-13 | 2.04E-10 |
| ENSG00000132465 | JCHAIN | 3470.344204 | 14446.49145 | -2.05751868 | 9.20E-13 | 3.74E-10 |
| ENSG00000235217 | TSPY26P | 71.49091321 | 4.610310369 | 3.899400076 | 1.61E-12 | 6.12E-10 |
| ENSG00000133878 | DUSP26 | 1152.014475 | 234.5246344 | 2.297995307 | 1.87E-12 | 6.97E-10 |
| ENSG00000106772 | PRUNE2 | 124.0232208 | 19.77164263 | 2.637915576 | 2.66E-12 | 9.34E-10 |
| ENSG00000225361 | PPP1R26-AS1 | 204.8389241 | 49.33889722 | 2.047373201 | 3.64E-12 | 1.26E-09 |
| ENSG00000111331 | OAS3 | 273.9642149 | 35.92931664 | 2.918621199 | 5.57E-12 | 1.89E-09 |
| ENSG00000106665 | CLIP2 | 572.6828272 | 93.07637591 | 2.612955916 | 6.19E-12 | 2.06E-09 |
| ENSG00000056558 | TRAF1 | 840.0135359 | 192.2852897 | 2.123020001 | 7.19E-12 | 2.35E-09 |
| ENSG00000004478 | FKBP4 | 1417.594694 | 6461.035415 | -2.188649415 | 9.10E-12 | 2.92E-09 |
| ENSG00000174885 | NLRP6 | 125.6734024 | 26.61558249 | 2.235446449 | 1.03E-11 | 3.26E-09 |
| ENSG00000186603 | HPDL | 192.4785505 | 939.9206088 | -2.288986138 | 4.82E-11 | 1.32E-08 |
| ENSG00000109971 | HSPA8 | 9969.324097 | 44989.81037 | -2.17402873 | 1.78E-10 | 4.39E-08 |
| ENSG00000130755 | GMFG | 302.7391358 | 64.47485132 | 2.22587719 | 1.83E-10 | 4.46E-08 |
| ENSG00000179873 | NLRP11 | 89.05902223 | 15.64658427 | 2.494040621 | 2.05E-10 | 4.93E-08 |
| ENSG00000145555 | MYO10 | 69.74476925 | 8.37977909 | 3.065737233 | 2.08E-10 | 4.93E-08 |
| ENSG00000130433 | CACNG6 | 936.179729 | 219.3373423 | 2.090416614 | 4.28E-10 | 9.49E-08 |
| ENSG00000167995 | BEST1 | 315.572077 | 36.83103021 | 3.08177858 | 4.71E-10 | 1.01E-07 |
| ENSG00000196139 | AKR1C3 | 172.8240107 | 42.00120532 | 2.036019796 | 5.84E-10 | 1.23E-07 |
| ENSG00000213654 | GPSM3 | 661.9226504 | 162.4301738 | 2.024525496 | 9.51E-10 | 1.93E-07 |
| ENSG00000105327 | BBC3 | 1101.847837 | 273.5464561 | 2.00705681 | 1.30E-09 | 2.49E-07 |
| ENSG00000162512 | SDC3 | 54.37104552 | 0.952347971 | 5.71881007 | 1.36E-09 | 2.59E-07 |
| ENSG00000226985 | LINC01203 | 72.50060184 | 0.64308525 | 6.705991759 | 1.45E-09 | 2.71E-07 |
| ENSG00000143318 | CASQ1 | 204.5888218 | 33.57929185 | 2.587370498 | 1.78E-09 | 3.28E-07 |
| ENSG00000182732 | RGS6 | 168.3514976 | 33.71896903 | 2.302718427 | 1.91E-09 | 3.45E-07 |
| ENSG00000211890 | IGHA2 | 92.98944246 | 10.9738872 | 3.050449475 | 1.98E-09 | 3.51E-07 |
| ENSG00000139269 | INHBE | 4642.862211 | 765.6477426 | 2.59930409 | 3.55E-09 | 5.69E-07 |
| ENSG00000233901 | LINC01503 | 133.1610391 | 32.64533679 | 2.017930335 | 4.33E-09 | 6.82E-07 |
| ENSG00000128573 | FOXP2 | 116.9197746 | 18.28620095 | 2.657959549 | 4.61E-09 | 7.15E-07 |
| ENSG00000243364 | EFNA4 | 53.87500584 | 244.643013 | -2.192033911 | 5.88E-09 | 8.89E-07 |
| ENSG00000105246 | EBI3 | 144.9450944 | 23.76063485 | 2.588659244 | 6.57E-09 | 9.76E-07 |
| ENSG00000035664 | DAPK2 | 98.41079552 | 21.57041765 | 2.175906296 | 8.10E-09 | 1.18E-06 |
| ENSG00000233191 | AC006372.2 | 79.13608663 | 16.79179967 | 2.226670234 | 8.23E-09 | 1.18E-06 |
| ENSG00000171428 | NAT1 | 25.66797997 | 106.8116308 | -2.0425446 | 9.38E-09 | 1.31E-06 |
| ENSG00000116761 | CTH | 1437.273734 | 352.971745 | 2.02357052 | 1.52E-08 | 2.03E-06 |
| ENSG00000114315 | HES1 | 42.09249261 | 2.097563368 | 4.325511778 | 1.96E-08 | 2.56E-06 |
| ENSG00000115956 | PLEK | 204.7919752 | 36.09203885 | 2.487486684 | 2.10E-08 | 2.72E-06 |
| ENSG00000253651 | SOD1P3 | 60.16151297 | 366.7547339 | -2.596342618 | 2.14E-08 | 2.75E-06 |
| ENSG00000215386 | MIR99AHG | 87.86537873 | 16.52118218 | 2.408160988 | 2.29E-08 | 2.88E-06 |
| ENSG00000172243 | CLEC7A | 37.32968304 | 165.3468186 | -2.152856098 | 2.33E-08 | 2.92E-06 |
| ENSG00000068976 | PYGM | 256.4030941 | 49.30083347 | 2.364177079 | 2.43E-08 | 3.02E-06 |
| ENSG00000155465 | SLC7A7 | 305.7838939 | 61.17387839 | 2.31001777 | 3.25E-08 | 3.85E-06 |
| ENSG00000100253 | MIOX | 40.51378082 | 0.952347971 | 5.289547599 | 4.40E-08 | 5.02E-06 |
| ENSG00000230943 | LINC02541 | 79.58591249 | 15.63249884 | 2.328247934 | 4.76E-08 | 5.37E-06 |
| ENSG00000197653 | DNAH10 | 42.26292753 | 3.801710206 | 3.474819027 | 4.97E-08 | 5.54E-06 |
| ENSG00000007516 | BAIAP3 | 71.78805987 | 11.07130809 | 2.688624679 | 5.08E-08 | 5.61E-06 |
| ENSG00000227507 | LTB | 281.9170863 | 58.1079003 | 2.28064982 | 5.75E-08 | 6.14E-06 |
| ENSG00000198400 | NTRK1 | 60.55528623 | 8.647603868 | 2.770282123 | 5.99E-08 | 6.29E-06 |
| ENSG00000114251 | WNT5A | 2205.309539 | 547.0827027 | 2.010197644 | 6.04E-08 | 6.31E-06 |
| ENSG00000123342 | MMP19 | 40.08851838 | 3.014877149 | 3.6906349 | 6.22E-08 | 6.46E-06 |
| ENSG00000250509 | AC034213.1 | 38.08240499 | 2.32267228 | 3.979545085 | 7.73E-08 | 7.76E-06 |
| ENSG00000100867 | DHRS2 | 36.66466918 | 1.929255751 | 4.155075741 | 8.37E-08 | 8.27E-06 |
| ENSG00000085514 | PILRA | 114.1037529 | 22.89651131 | 2.291667921 | 1.06E-07 | 1.03E-05 |
| ENSG00000167895 | TMC8 | 88.08190938 | 21.22250969 | 2.040473587 | 1.18E-07 | 1.12E-05 |
| ENSG00000105649 | RAB3A | 7.810750846 | 73.16296702 | -3.257823554 | 1.31E-07 | 1.23E-05 |
| ENSG00000100055 | CYTH4 | 36.00790958 | 3.158624956 | 3.519790408 | 1.47E-07 | 1.35E-05 |
| ENSG00000070808 | CAMK2A | 42.74858797 | 0.66764506 | 5.949572662 | 1.51E-07 | 1.36E-05 |
| ENSG00000230666 | CEACAM22P | 36.05911886 | 2.396351708 | 3.889823326 | 1.67E-07 | 1.49E-05 |
| ENSG00000173156 | RHOD | 72.99178402 | 9.812490102 | 2.918550095 | 1.71E-07 | 1.52E-05 |
| ENSG00000152463 | OLAH | 44.61643404 | 6.675632252 | 2.751532232 | 2.17E-07 | 1.87E-05 |
| ENSG00000172232 | AZU1 | 46.68756104 | 0.33382253 | 6.953473423 | 2.40E-07 | 2.04E-05 |
| ENSG00000113504 | SLC12A7 | 40.37670402 | 5.087880707 | 2.969567689 | 2.97E-07 | 2.39E-05 |
| ENSG00000102287 | GABRE | 23.64101268 | 99.58591176 | -2.058696752 | 3.27E-07 | 2.61E-05 |
| ENSG00000203497 | PDCD4-AS1 | 114.389206 | 25.72131374 | 2.131300039 | 3.50E-07 | 2.75E-05 |
| ENSG00000163431 | LMOD1 | 84.52006309 | 20.50656336 | 2.022598133 | 4.04E-07 | 3.12E-05 |
| ENSG00000163121 | NEURL3 | 40.5870487 | 5.038761088 | 2.979197152 | 5.41E-07 | 4.16E-05 |
| ENSG00000227471 | AKR1B15 | 66.39301159 | 11.07619706 | 2.540799512 | 6.32E-07 | 4.79E-05 |
| ENSG00000176490 | DIRAS1 | 33.53363543 | 1.394884118 | 4.584093448 | 6.45E-07 | 4.85E-05 |
| ENSG00000205436 | EXOC3L4 | 52.44989354 | 7.909890429 | 2.683540413 | 8.00E-07 | 5.83E-05 |
| ENSG00000130513 | GDF15 | 37.61302536 | 2.740648618 | 3.752850877 | 1.36E-06 | 8.94E-05 |
| ENSG00000128596 | CCDC136 | 60.58928024 | 14.06034751 | 2.132933842 | 2.17E-06 | 0.0001322 |
| ENSG00000274020 | LINC01138 | 81.71437805 | 17.95796382 | 2.176939857 | 2.16E-06 | 0.0001322 |
| ENSG00000166793 | YPEL4 | 86.43332648 | 12.53835685 | 2.745661088 | 2.63E-06 | 0.0001533 |
| ENSG00000248738 | AC037441.1 | 35.86439076 | 4.110972927 | 3.120776702 | 3.09E-06 | 0.0001723 |
| ENSG00000163683 | SMIM14 | 117.3174709 | 27.60099029 | 2.076680172 | 3.25E-06 | 0.0001792 |
| ENSG00000138131 | LOXL4 | 87.48554778 | 19.73788636 | 2.120043107 | 3.24E-06 | 0.0001792 |
| ENSG00000111254 | AKAP3 | 47.13274293 | 9.364368546 | 2.300504845 | 3.56E-06 | 0.0001949 |
| ENSG00000133216 | EPHB2 | 49.70762341 | 7.811651191 | 2.619381343 | 3.58E-06 | 0.0001952 |
| ENSG00000144120 | TMEM177 | 98.14744351 | 434.098389 | -2.145877589 | 3.66E-06 | 0.0001991 |
| ENSG00000162817 | C1orf115 | 11.72210106 | 55.71620071 | -2.276077437 | 3.72E-06 | 0.000202 |
| ENSG00000130203 | APOE | 451.2851935 | 92.9271435 | 2.272930743 | 8.00E-06 | 0.0003769 |
| ENSG00000263874 | LINC00672 | 55.39449597 | 12.03494879 | 2.176314817 | 8.52E-06 | 0.0003983 |
| ENSG00000197406 | DIO3 | 27.57508573 | 1.95381556 | 3.742106546 | 8.74E-06 | 0.0004076 |
| ENSG00000245573 | BDNF-AS | 46.17059659 | 8.397935146 | 2.441010145 | 8.94E-06 | 0.0004134 |
| ENSG00000211900 | IGHJ6 | 8.698058961 | 40.7383167 | -2.237472939 | 9.11E-06 | 0.0004206 |
| ENSG00000144642 | RBMS3 | 50.72766293 | 11.45145753 | 2.127740382 | 9.45E-06 | 0.0004294 |
| ENSG00000175868 | CALCB | 38.49876312 | 5.822801442 | 2.669925226 | 1.15E-05 | 0.0004965 |
| ENSG00000129654 | FOXJ1 | 40.51011384 | 7.185444074 | 2.474658273 | 1.17E-05 | 0.0005026 |
| ENSG00000280870 | MIR325HG | 48.26531001 | 9.68003502 | 2.31916746 | 1.23E-05 | 0.0005185 |
| ENSG00000242759 | LINC00882 | 27.12498965 | 2.656494809 | 3.296857087 | 1.62E-05 | 0.0006427 |
| ENSG00000188158 | NHS | 28.97285958 | 3.742116207 | 2.93893843 | 1.64E-05 | 0.0006485 |
| ENSG00000112297 | CRYBG1 | 39.75644325 | 6.88386303 | 2.488148413 | 1.67E-05 | 0.0006518 |
| ENSG00000132681 | ATP1A4 | 94.2062642 | 20.46873647 | 2.184351019 | 1.98E-05 | 0.0007461 |
| ENSG00000162496 | DHRS3 | 76.5242094 | 16.21960114 | 2.217048397 | 2.36E-05 | 0.0008595 |
| ENSG00000235592 | AC004674.1 | 18.03014051 | 0 | 6.552020012 | 2.42E-05 | 0.0008789 |
| ENSG00000260337 | AC091544.4 | 76.58143834 | 17.67535718 | 2.090891834 | 2.68E-05 | 0.0009479 |
| ENSG00000169064 | ZBBX | 39.93581083 | 8.461140195 | 2.221064933 | 2.73E-05 | 0.0009602 |
| ENSG00000168070 | MAJIN | 27.23567663 | 2.990317339 | 3.133310452 | 2.75E-05 | 0.0009643 |
| ENSG00000183305 | MAGEA2B | 27.52047969 | 174.9620404 | -2.656427924 | 2.93E-05 | 0.001003 |
| ENSG00000267769 | AC011498.6 | 86.84761632 | 16.35694519 | 2.385864299 | 2.91E-05 | 0.001003 |
| ENSG00000205015 | LINC02138 | 12.55085176 | 0 | 6.012269915 | 2.99E-05 | 0.0010161 |
| ENSG00000244383 | FAM3D-AS1 | 40.90162416 | 9.6905094 | 2.070555413 | 3.02E-05 | 0.0010227 |
| ENSG00000163734 | CXCL3 | 9.247798558 | 43.18949535 | -2.233484831 | 3.22E-05 | 0.001078 |
| ENSG00000164692 | COL1A2 | 34.7082089 | 7.009454781 | 2.318025516 | 3.30E-05 | 0.0010906 |
| ENSG00000167711 | SERPINF2 | 28.86404152 | 2.881603721 | 3.249767525 | 3.34E-05 | 0.0010989 |
| ENSG00000172349 | IL16 | 32.21091334 | 4.143214412 | 2.890296671 | 3.42E-05 | 0.0011186 |
| ENSG00000179141 | MTUS2-AS1 | 33.32238464 | 3.334614249 | 3.286075852 | 3.46E-05 | 0.0011238 |
| ENSG00000126353 | CCR7 | 23.32800758 | 1.812860457 | 3.714289132 | 3.61E-05 | 0.0011582 |
| ENSG00000183160 | TMEM119 | 23.88693589 | 1.880136132 | 3.558111068 | 4.32E-05 | 0.0013382 |
| ENSG00000168427 | KLHL30 | 26.75460346 | 3.60884278 | 2.832494604 | 4.73E-05 | 0.0014321 |
| ENSG00000182601 | HS3ST4 | 31.76911403 | 5.646812149 | 2.451073166 | 4.79E-05 | 0.0014479 |
| ENSG00000137474 | MYO7A | 45.22381076 | 10.66101343 | 2.060477061 | 5.20E-05 | 0.0015388 |
| ENSG00000147434 | CHRNA6 | 36.15168446 | 6.029754297 | 2.563915524 | 5.24E-05 | 0.0015464 |
| ENSG00000105550 | FGF21 | 12.32487634 | 0 | 5.990379654 | 5.69E-05 | 0.0016557 |
| ENSG00000100985 | MMP9 | 20.57653455 | 1.479037927 | 3.851128654 | 6.37E-05 | 0.0018236 |
| ENSG00000271743 | AF287957.1 | 19.04901787 | 1.370324309 | 3.777782332 | 6.49E-05 | 0.001844 |
| ENSG00000198156 | NPIPB6 | 30.49578899 | 5.730965957 | 2.380364342 | 7.01E-05 | 0.0019384 |
| ENSG00000121552 | CSTA | 10.90161879 | 0 | 5.816863797 | 7.31E-05 | 0.0019966 |
| ENSG00000200087 | SNORA73B | 9.304515397 | 40.20091551 | -2.109307138 | 7.92E-05 | 0.0021108 |
| ENSG00000041982 | TNC | 24.17329513 | 3.541567105 | 2.784603564 | 7.96E-05 | 0.0021144 |
| ENSG00000279821 | AC145098.2 | 129.7280296 | 30.47024609 | 2.069974564 | 8.34E-05 | 0.0021845 |
| ENSG00000207827 | MIR30A | 16.42478286 | 0.417976338 | 5.453332712 | 8.63E-05 | 0.0022371 |
| ENSG00000132837 | DMGDH | 23.06461305 | 2.982635663 | 3.011043772 | 9.09E-05 | 0.0023234 |
| ENSG00000069431 | ABCC9 | 82.8545068 | 17.54441688 | 2.231781678 | 9.24E-05 | 0.0023431 |
| ENSG00000272666 | U62317.1 | 20.19327849 | 1.31073031 | 3.868893209 | 9.26E-05 | 0.0023447 |
| ENSG00000234936 | AC010883.1 | 55.6146936 | 12.30556627 | 2.143523007 | 9.33E-05 | 0.0023578 |
| ENSG00000137101 | CD72 | 19.14775527 | 1.370324309 | 3.777002555 | 9.55E-05 | 0.0023969 |
| ENSG00000234043 | NUDT9P1 | 31.61550037 | 6.374051207 | 2.282453365 | 0.0001021 | 0.0025254 |
| ENSG00000270124 | AC092127.2 | 20.53544845 | 1.704146839 | 3.561822196 | 0.0001069 | 0.002606 |
| ENSG00000229314 | ORM1 | 15.74413826 | 0.33382253 | 5.376700222 | 0.0001097 | 0.0026478 |
| ENSG00000129951 | PLPPR3 | 39.77418479 | 2.498661573 | 3.928974569 | 0.0001113 | 0.0026825 |
| ENSG00000233359 | AC114485.1 | 0.826910118 | 16.42294295 | -4.202166456 | 0.0001142 | 0.0027466 |
| ENSG00000145242 | EPHA5 | 2.312406241 | 21.22123177 | -3.147075858 | 0.0001166 | 0.002796 |
| ENSG00000186818 | LILRB4 | 23.83756719 | 3.348699678 | 2.792038409 | 0.0001283 | 0.0030087 |
| ENSG00000197540 | GZMM | 57.5788006 | 10.83700272 | 2.378294937 | 0.000134 | 0.0030993 |
| ENSG00000270441 | AC135506.1 | 20.46218058 | 0.927788161 | 4.325026262 | 0.0001521 | 0.0034449 |
| ENSG00000141934 | PLPP2 | 96.97196392 | 19.27486103 | 2.304695224 | 0.0001574 | 0.0035285 |
| ENSG00000237541 | HLA-DQA2 | 22.35299136 | 3.014877149 | 2.849794945 | 0.0001598 | 0.0035485 |
| ENSG00000158113 | LRRC43 | 30.56262901 | 6.113908106 | 2.299169469 | 0.0001665 | 0.0036617 |
| ENSG00000205221 | VIT | 19.27958056 | 1.98884975 | 3.232660371 | 0.0001731 | 0.0037611 |
| ENSG00000230615 | AL139220.2 | 24.9333794 | 4.110972927 | 2.578965912 | 0.0001792 | 0.0038448 |
| ENSG00000271344 | AC018638.6 | 62.00861475 | 11.84615199 | 2.347940136 | 0.0001839 | 0.0039168 |
| ENSG00000132692 | BCAN | 36.75053671 | 8.162351854 | 2.143820845 | 0.0002101 | 0.0043296 |
| ENSG00000242574 | HLA-DMB | 26.14446587 | 4.911891414 | 2.409451042 | 0.0002132 | 0.0043727 |
| ENSG00000204583 | LRCOL1 | 14.96659684 | 0.30926272 | 5.298342447 | 0.0002134 | 0.0043734 |
| ENSG00000137673 | MMP7 | 1.10408064 | 16.00007764 | -3.746809134 | 0.0002258 | 0.0045806 |
| ENSG00000235831 | BHLHE40-AS1 | 24.46149495 | 4.444795457 | 2.451473174 | 0.0002261 | 0.0045806 |
| ENSG00000274414 | AL121772.1 | 16.13750333 | 0.952347971 | 3.978392451 | 0.0002419 | 0.0048101 |
| ENSG00000276644 | DACH1 | 16.52810753 | 1.370324309 | 3.584307144 | 0.0002549 | 0.0050303 |
| ENSG00000099617 | EFNA2 | 9.299007835 | 0 | 5.575062258 | 0.0002576 | 0.005074 |
| ENSG00000122877 | EGR2 | 75.9446407 | 17.0153938 | 2.130150738 | 0.000261 | 0.0051233 |
| ENSG00000089250 | NOS1 | 0.276250232 | 12.2628504 | -5.216761299 | 0.000262 | 0.0051322 |
| ENSG00000159337 | PLA2G4D | 3.909509638 | 22.53475478 | -2.509021933 | 0.0002678 | 0.0052076 |
| ENSG00000117971 | CHRNB4 | 26.92660874 | 5.364901942 | 2.345276105 | 0.0002759 | 0.0053411 |
| ENSG00000169245 | CXCL10 | 8.153826926 | 34.43084473 | -2.05517979 | 0.0002966 | 0.0056405 |
| ENSG00000116701 | NCF2 | 31.25863413 | 6.900741163 | 2.162301472 | 0.0002969 | 0.0056407 |
| ENSG00000223475 | AC099681.1 | 27.92827082 | 6.089348296 | 2.178824699 | 0.0003025 | 0.0057119 |
| ENSG00000167874 | TMEM88 | 45.81530069 | 9.993368367 | 2.164954981 | 0.0003035 | 0.0057131 |
| ENSG00000137501 | SYTL2 | 56.26734968 | 13.76365543 | 2.021659902 | 0.0003165 | 0.0058908 |
| ENSG00000005243 | COPZ2 | 16.02681635 | 1.2861705 | 3.555658619 | 0.0003314 | 0.0061053 |
| ENSG00000278390 | AL354696.2 | 32.03525526 | 6.806112975 | 2.211281829 | 0.0003349 | 0.0061509 |
| ENSG00000272382 | AC025171.5 | 19.20080512 | 1.763740838 | 3.446227734 | 0.0003354 | 0.006154 |
| ENSG00000254536 | AL360181.3 | 16.51431737 | 1.31073031 | 3.589279478 | 0.000358 | 0.0064906 |
| ENSG00000115896 | PLCL1 | 29.41743393 | 6.78434587 | 2.14393488 | 0.0003587 | 0.0064976 |
| ENSG00000122176 | FMOD | 16.01854792 | 1.394884118 | 3.514977101 | 0.0003634 | 0.0065705 |
| ENSG00000248099 | INSL3 | 23.43593369 | 3.29958006 | 2.771193765 | 0.0003736 | 0.006727 |
| ENSG00000285799 | AL645929.2 | 20.06170925 | 2.431385897 | 3.056707475 | 0.0003765 | 0.0067668 |
| ENSG00000259306 | AC020891.2 | 15.52368458 | 1.370324309 | 3.480328186 | 0.0003912 | 0.0069498 |
| ENSG00000237988 | OR2I1P | 27.62720113 | 5.671371958 | 2.244520183 | 0.0004098 | 0.0071878 |
| ENSG00000111961 | SASH1 | 39.74016244 | 8.478018328 | 2.246120154 | 0.0004141 | 0.0072429 |
| ENSG00000206105 | KRTAP20-4 | 21.09528289 | 3.576601294 | 2.591066862 | 0.0004176 | 0.0072619 |
| ENSG00000228278 | ORM2 | 35.60721055 | 7.242245369 | 2.254232078 | 0.0004194 | 0.0072792 |
| ENSG00000182584 | ACTL10 | 22.98677207 | 93.63810724 | -2.047867225 | 0.0004398 | 0.0075675 |
| ENSG00000284128 | AP000356.3 | 57.59414694 | 12.24236122 | 2.206489201 | 0.0004421 | 0.0075872 |
| ENSG00000157927 | RADIL | 36.40589027 | 5.640408395 | 2.630468844 | 0.0004471 | 0.0076441 |
| ENSG00000173210 | ABLIM3 | 21.47395167 | 3.766676017 | 2.494246127 | 0.0004563 | 0.0077436 |
| ENSG00000260498 | AC126696.3 | 41.8720531 | 6.926578896 | 2.539414925 | 0.0004605 | 0.007808 |
| ENSG00000227060 | LINC00629 | 20.59491199 | 3.541567105 | 2.555605792 | 0.0004611 | 0.0078099 |
| ENSG00000258647 | LINC00930 | 16.40088369 | 1.679587029 | 3.243911602 | 0.0004854 | 0.008131 |
| ENSG00000197077 | KIAA1671 | 28.13768103 | 6.309568236 | 2.2047793 | 0.0004927 | 0.008223 |
| ENSG00000204420 | MPIG6B | 27.5241325 | 2.26307828 | 3.560207428 | 0.000495 | 0.0082475 |
| ENSG00000248746 | ACTN3 | 43.81316704 | 8.099965151 | 2.3937485 | 0.0005033 | 0.0083694 |
| ENSG00000235621 | LINC00494 | 23.08759193 | 3.166306632 | 2.799991771 | 0.0005105 | 0.0084519 |
| ENSG00000100298 | APOBEC3H | 26.02366988 | 5.280748133 | 2.297276064 | 0.0005217 | 0.008584 |
| ENSG00000165178 | NCF1C | 15.63345128 | 1.261610691 | 3.522433878 | 0.0005272 | 0.00865 |
| ENSG00000263004 | AC007114.1 | 34.22043765 | 8.488492708 | 2.008863496 | 0.000543 | 0.0088382 |
| ENSG00000166111 | SVOP | 28.96920677 | 5.513538721 | 2.342588194 | 0.0005492 | 0.0089144 |
| ENSG00000226472 | AC008013.1 | 17.48933358 | 1.812860457 | 3.295089234 | 0.0005489 | 0.0089144 |
| ENSG00000231217 | AC097347.1 | 8.153826926 | 0 | 5.406471262 | 0.0005721 | 0.009188 |
| ENSG00000120051 | CFAP58 | 22.91468054 | 4.911891414 | 2.230855601 | 0.0006616 | 0.0102736 |
| ENSG00000211895 | IGHA1 | 17.70059855 | 0.927788161 | 4.121085111 | 0.0006733 | 0.010394 |
| ENSG00000258881 | AC007040.2 | 2.039837165 | 16.2187828 | -2.952946243 | 0.0006822 | 0.0104691 |
| ENSG00000100122 | CRYBB1 | 17.44364604 | 2.431385897 | 2.830546342 | 0.0006904 | 0.0105858 |
| ENSG00000082397 | EPB41L3 | 2.424013515 | 16.29246222 | -2.745434579 | 0.0007534 | 0.0113927 |
| ENSG00000086506 | HBQ1 | 8.305614181 | 0 | 5.406756698 | 0.0007718 | 0.0115524 |
| ENSG00000276509 | AC239799.2 | 27.14887465 | 6.423170826 | 2.063356858 | 0.000856 | 0.0124003 |
| ENSG00000234981 | AC244034.1 | 29.185059 | 6.363576827 | 2.166592319 | 0.0008834 | 0.0127036 |
| ENSG00000283959 | AP002851.1 | 20.78503865 | 2.631935 | 2.920540603 | 0.0009672 | 0.0136819 |
| ENSG00000167654 | ATCAY | 21.00547815 | 2.96575753 | 2.76726542 | 0.001008 | 0.0141601 |
| ENSG00000124006 | OBSL1 | 46.38552853 | 10.86924421 | 2.058047508 | 0.0010157 | 0.0142359 |
| ENSG00000259405 | ISCA1P4 | 40.82562376 | 9.48076384 | 2.065119386 | 0.0010192 | 0.0142629 |
| ENSG00000267938 | EIF1P6 | 27.63640402 | 5.90695525 | 2.180162971 | 0.0010292 | 0.0143708 |
| ENSG00000117322 | CR2 | 3.972668502 | 22.30406046 | -2.438204668 | 0.0010458 | 0.0145361 |
| ENSG00000183654 | Mar-11 | 18.65381222 | 2.673372943 | 2.870351878 | 0.001058 | 0.0146349 |
| ENSG00000232394 | AC090696.1 | 17.76650411 | 2.371791898 | 2.867158463 | 0.0010649 | 0.0146664 |
| ENSG00000166482 | MFAP4 | 12.44935349 | 0.64308525 | 4.1883413 | 0.001076 | 0.0147869 |
| ENSG00000261578 | AP003119.3 | 31.99691585 | 7.256330799 | 2.106285427 | 0.001079 | 0.0148058 |
| ENSG00000142185 | TRPM2 | 29.38620079 | 6.181183781 | 2.196364854 | 0.0010859 | 0.0148779 |
| ENSG00000168060 | NAALADL1 | 23.82652372 | 4.669904368 | 2.316386482 | 0.0010929 | 0.014951 |
| ENSG00000101194 | SLC17A9 | 14.86141742 | 1.595433221 | 3.129796856 | 0.0011139 | 0.0150811 |
| ENSG00000273007 | AC021205.3 | 12.22339225 | 0.64308525 | 4.153511249 | 0.0011346 | 0.0152933 |
| ENSG00000133067 | LGR6 | 10.72502625 | 0.33382253 | 4.816063666 | 0.0011412 | 0.0153364 |
| ENSG00000246130 | AC107959.2 | 17.23055467 | 2.656494809 | 2.648030752 | 0.0011983 | 0.0158828 |
| ENSG00000111644 | ACRBP | 15.53288747 | 1.345764499 | 3.495003604 | 0.0012416 | 0.0163029 |
| ENSG00000272986 | AC009570.1 | 3.633259405 | 21.81950489 | -2.566544787 | 0.0013285 | 0.0171965 |
| ENSG00000285159 | AL627422.2 | 20.41647887 | 4.075938737 | 2.295957081 | 0.0013967 | 0.0179397 |
| ENSG00000070388 | FGF22 | 23.31448764 | 4.588543264 | 2.363051548 | 0.0015814 | 0.0196218 |
| ENSG00000168334 | XIRP1 | 20.59583228 | 4.803177796 | 2.090079193 | 0.0016763 | 0.0205207 |
| ENSG00000168928 | CTRB2 | 9.41888354 | 0.30926272 | 4.643461582 | 0.0016825 | 0.0205694 |
| ENSG00000261441 | AC124068.2 | 2.638945462 | 17.12352593 | -2.748719983 | 0.0018762 | 0.0223559 |
| ENSG00000196876 | SCN8A | 17.74562185 | 2.873922045 | 2.665678009 | 0.0018776 | 0.0223559 |
| ENSG00000280087 | AC011481.3 | 11.73497094 | 0.66764506 | 4.088631385 | 0.0019337 | 0.0228906 |
| ENSG00000068831 | RASGRP2 | 15.86676066 | 2.765208427 | 2.524765687 | 0.0020992 | 0.02434 |
| ENSG00000204758 | AC008429.1 | 26.27353029 | 5.52762415 | 2.20605946 | 0.0022028 | 0.0251626 |
| ENSG00000008118 | CAMK1G | 20.02770107 | 3.166306632 | 2.586019412 | 0.0022095 | 0.0251926 |
| ENSG00000134539 | KLRD1 | 20.16228723 | 4.778617986 | 2.069833065 | 0.0023075 | 0.0260492 |
| ENSG00000141161 | UNC45B | 11.78616604 | 0.952347971 | 3.541031098 | 0.0023753 | 0.0266998 |
| ENSG00000149527 | PLCH2 | 4.247078156 | 20.15097372 | -2.199192437 | 0.0024561 | 0.0272395 |
| ENSG00000237877 | LINC01473 | 14.24943925 | 1.570873411 | 3.079542651 | 0.0024724 | 0.02737 |
| ENSG00000152292 | SH2D6 | 17.3375605 | 3.517007295 | 2.299923272 | 0.0024864 | 0.0274583 |
| ENSG00000258947 | TUBB3 | 21.69256478 | 4.91957309 | 2.09234943 | 0.0026488 | 0.0287822 |
| ENSG00000243276 | AC068633.1 | 11.51451726 | 1.085621398 | 3.453500003 | 0.0026851 | 0.0291097 |
| ENSG00000164707 | SLC13A4 | 20.6737016 | 3.991784929 | 2.349143931 | 0.0028374 | 0.0303801 |
| ENSG00000230333 | AC004160.1 | 13.67975186 | 1.00146759 | 3.740266318 | 0.0028449 | 0.0304433 |
| ENSG00000284664 | AL161756.3 | 16.63603365 | 3.408293677 | 2.276970892 | 0.0028552 | 0.0304991 |
| ENSG00000245164 | LINC00861 | 165.2533287 | 995.9162406 | -2.589873433 | 0.0028836 | 0.030749 |
| ENSG00000115596 | WNT6 | 11.11839132 | 0.952347971 | 3.444211993 | 0.0029952 | 0.0317164 |
| ENSG00000263327 | TAPT1-AS1 | 20.7742654 | 5.112440516 | 2.018276051 | 0.0031572 | 0.0327864 |
| ENSG00000239322 | ATP6V1B1-AS1 | 0.660426582 | 10.2558446 | -4.040600565 | 0.0032442 | 0.0333867 |
| ENSG00000279339 | AC100788.2 | 22.76289329 | 5.481297236 | 2.057330444 | 0.0032774 | 0.0335765 |
| ENSG00000137868 | STRA6 | 16.40180398 | 2.523221382 | 2.613847587 | 0.0033385 | 0.0340155 |
| ENSG00000251143 | AP002490.1 | 22.47929491 | 5.179716191 | 2.075624722 | 0.0034432 | 0.0345583 |
| ENSG00000259172 | AC023024.1 | 13.37868217 | 2.097563368 | 2.672223344 | 0.0034503 | 0.0346111 |
| ENSG00000185215 | TNFAIP2 | 18.11786279 | 4.184652355 | 2.11248043 | 0.0035755 | 0.0355048 |
| ENSG00000070886 | EPHA8 | 12.71825558 | 1.237050882 | 3.248400941 | 0.0035822 | 0.0355249 |
| ENSG00000100368 | CSF2RB | 4.077833752 | 17.51903872 | -2.073339305 | 0.0036246 | 0.0358674 |
| ENSG00000237979 | AC007389.4 | 13.33298045 | 1.897014265 | 2.901293872 | 0.0036314 | 0.0358957 |
| ENSG00000237596 | AL138828.1 | 10.74524427 | 1.061061589 | 3.369779312 | 0.0036301 | 0.0358957 |
| ENSG00000007314 | SCN4A | 13.10151164 | 1.64455284 | 2.934140933 | 0.003708 | 0.0363532 |
| ENSG00000251615 | AC104825.1 | 32.18336135 | 7.369115043 | 2.076066264 | 0.0037299 | 0.0364946 |
| ENSG00000110446 | SLC15A3 | 15.40749003 | 2.656494809 | 2.483199451 | 0.0037544 | 0.0366553 |
| ENSG00000225931 | AL139246.2 | 12.17770471 | 1.704146839 | 2.824389893 | 0.0037914 | 0.036853 |
| ENSG00000184032 | KRTAP20-2 | 23.9353843 | 5.406339885 | 2.199493292 | 0.0039024 | 0.0375753 |
| ENSG00000105219 | CNTD2 | 30.35872636 | 7.179040321 | 2.050177915 | 0.0039208 | 0.037676 |
| ENSG00000247157 | LINC01252 | 10.62536856 | 0.97690778 | 3.373046006 | 0.0039269 | 0.0376953 |
| ENSG00000248213 | CICP16 | 36.84427865 | 7.635661898 | 2.260347028 | 0.0039562 | 0.0378972 |
| ENSG00000265091 | AP001496.2 | 0.552500465 | 9.307567251 | -3.968173535 | 0.0039658 | 0.0379376 |
| ENSG00000229672 | AL450322.2 | 12.23166068 | 1.419443928 | 3.133594352 | 0.0039677 | 0.0379376 |
| ENSG00000124257 | NEURL2 | 13.48660828 | 2.062529178 | 2.68857652 | 0.0040484 | 0.0384775 |
| ENSG00000277496 | AL357033.4 | 3.584810995 | 21.40292838 | -2.525515417 | 0.0041309 | 0.0389941 |
| ENSG00000171631 | P2RY6 | 4.128122741 | 18.05702141 | -2.16499659 | 0.0041381 | 0.0389941 |
| ENSG00000125148 | MT2A | 4.293685988 | 18.54296986 | -2.103326291 | 0.0042366 | 0.0395665 |
| ENSG00000197558 | SSPO | 18.55875598 | 4.016344738 | 2.177603352 | 0.0042504 | 0.0396757 |
| ENSG00000275772 | AC244157.2 | 15.79901452 | 3.492447486 | 2.175382382 | 0.0042955 | 0.0399534 |
| ENSG00000215034 | DSTNP4 | 10.02626027 | 0.66764506 | 3.867713716 | 0.004314 | 0.0401052 |
| ENSG00000127249 | ATP13A4 | 14.04737718 | 2.29811247 | 2.568016554 | 0.0043294 | 0.0402077 |
| ENSG00000262152 | LINC00514 | 8.902881899 | 0.33382253 | 4.540322755 | 0.004421 | 0.0407475 |
| ENSG00000266990 | AC004528.1 | 12.98439681 | 1.237050882 | 3.274867978 | 0.0044714 | 0.041047 |
| ENSG00000204790 | AL163540.1 | 15.69108841 | 3.383733868 | 2.187110332 | 0.0044852 | 0.0411323 |
| ENSG00000183230 | CTNNA3 | 12.73663302 | 1.345764499 | 3.22933232 | 0.0048018 | 0.0430705 |
| ENSG00000182578 | CSF1R | 11.62060279 | 1.704146839 | 2.756118 | 0.0048632 | 0.0435071 |
| ENSG00000177363 | LRRN4CL | 18.79207954 | 3.893545691 | 2.22669147 | 0.0049806 | 0.0442482 |
| ENSG00000151012 | SLC7A11 | 1882.502404 | 390.7185812 | 2.267945458 | 0.0052737 | 0.0459611 |
| ENSG00000105697 | HAMP | 23.55855609 | 5.404825103 | 2.065120151 | 0.0053604 | 0.0464379 |
| ENSG00000279853 | AC004453.2 | 11.02517566 | 0.927788161 | 3.467352327 | 0.0053773 | 0.0465346 |
| ENSG00000232044 | LINC01105 | 2.147763283 | 12.51810453 | -2.541044667 | 0.0054397 | 0.0468525 |
| ENSG00000251301 | LINC02384 | 18.16263004 | 4.46167359 | 2.065646137 | 0.0054484 | 0.0469057 |
| ENSG00000130518 | IQCN | 14.47081322 | 2.213958662 | 2.618842535 | 0.0056206 | 0.0480234 |
| ENSG00000270276 | HIST2H4B | 11.39740242 | 1.788300647 | 2.694879176 | 0.0057058 | 0.048482 |
| ENSG00000124772 | CPNE5 | 12.50056277 | 1.929255751 | 2.624172913 | 0.005777 | 0.0487961 |
| ENSG00000108551 | RASD1 | 9.914667164 | 0.618525441 | 3.873601592 | 0.0057775 | 0.0487961 |
| ENSG00000211893 | IGHG2 | 15.68742142 | 2.189398852 | 2.752687573 | 0.0058881 | 0.0494562 |
| ENSG00000279432 | AC015799.1 | 12.90403685 | 1.345764499 | 3.249665538 | 0.0058863 | 0.0494562 |
| ENSG00000249359 | AC093274.1 | 11.57215438 | 1.419443928 | 3.054550092 | 0.0059193 | 0.0496723 |
| ENSG00000224243 | SOX1-OT | 7.814432002 | 0.417976338 | 4.359166636 | 0.005932 | 0.0497104 |

**Supplementary Table 2.** Full list of DNA damage/ DNA repair compound library (160 compounds)

| Compound Name | Target |
| --- | --- |
| Veliparib (ABT-888) | PARP |
| Olaparib (AZD2281, Ku-0059436) | PARP |
| Iniparib (BSI-201) | PARP |
| KU-55933 (ATM Kinase Inhibitor) | ATM/ATR |
| Rucaparib (AG-014699,PF-01367338) phosphate | PARP |
| INO-1001 (3-Aminobenzamide) | PARP |
| Capecitabine | DNA/RNA Synthesis |
| BIBR 1532 | Telomerase |
| Raltitrexed | DNA/RNA Synthesis |
| Cladribine | DNA/RNA Synthesis |
| Doxorubicin (Adriamycin) HCl | Topoisomerase |
| Fluorouracil (5-Fluoracil, 5-FU) | DNA/RNA Synthesis |
| Bendamustine HCl | DNA/RNA Synthesis |
| Nelarabine | DNA/RNA Synthesis |
| Clofarabine | DNA/RNA Synthesis |
| Dacarbazine | DNA/RNA Synthesis |
| Dexrazoxane HCl (ICRF-187, ADR-529) | Topoisomerase |
| Epirubicin HCl | Topoisomerase |
| Etoposide | Topoisomerase |
| Fludarabine Phosphate | DNA/RNA Synthesis |
| Topotecan HCl | Topoisomerase |
| Temozolomide | DNA/RNA Synthesis,Autophagy |
| Altretamine | DNA alkylator |
| Carmofur | DNA/RNA Synthesis |
| Floxuridine | DNA/RNA Synthesis |
| Tegafur (FT-207, NSC 148958) | DNA/RNA Synthesis |
| Ifosfamide | DNA/RNA Synthesis |
| Mercaptopurine (6-MP) | DNA/RNA Synthesis |
| Streptozotocin (STZ) | DNA alkylator |
| Costunolide | Telomerase |
| Flupirtine maleate | DNA/RNA Synthesis |
| Gatifloxacin | Topoisomerase |
| Amonafide | Topoisomerase |
| Daptomycin | Anti-infection,DNA/RNA Synthesis |
| Pirarubicin | Topoisomerase |
| Moxifloxacin HCl | Topoisomerase |
| KU-60019 | ATM/ATR |
| Busulfan | DNA alkylator |
| Gemcitabine | Autophagy,DNA/RNA Synthesis |
| Enoxacin | Topoisomerase |
| Rifapentine | DNA/RNA Synthesis |
| Rifampin | DNA/RNA Synthesis |
| Trifluridine | DNA/RNA Synthesis |
| Azacitidine | DNA Methyltransferase |
| Vidarabine | DNA/RNA Synthesis |
| Teniposide | Topoisomerase |
| Rifaximin | DNA/RNA Synthesis |
| Lomustine | DNA/RNA Synthesis |
| Hydroxyurea | DNA/RNA Synthesis |
| Nicotinamide (Vitamin B3) | Sirtuin |
| Metronidazole | DNA/RNA Synthesis |
| Levofloxacin | Topoisomerase |
| Cyclocytidine HCl | DNA/RNA Synthesis |
| Adenine HCl | DNA/RNA Synthesis |
| Uridine | DNA/RNA Synthesis |
| Cyclophosphamide Monohydrate | DNA alkylator |
| Balofloxacin | Topoisomerase |
| AG-14361 | PARP |
| A-966492 | PARP |
| Irinotecan HCl Trihydrate | Topoisomerase |
| (-)-Epigallocatechin Gallate | DNA Methyltransferase,HER2,  Telomerase,EGFR,Fatty Acid Synthase |
| Nalidixic acid | Topoisomerase |
| (S)-10-Hydroxycamptothecin | Topoisomerase |
| Mitoxantrone 2HCl | Topoisomerase |
| Novobiocin Sodium | Topoisomerase |
| Ciprofibrate | PPAR |
| Niraparib (MK-4827) | PARP |
| Sofosbuvir (PSI-7977, GS-7977) | DNA/RNA Synthesis |
| T0070907 | PPAR |
| GW9662 | PPAR |
| Clevudine | DNA/RNA Synthesis |
| Daunorubicin HCl | Topoisomerase |
| Flumequine | Topoisomerase |
| Betulinic acid | Topoisomerase |
| Carmustine | DNA/RNA Synthesis |
| Cycloastragenol | Telomerase |
| Hydroxy Camptothecine | Topoisomerase |
| Vitamin D2 | DNA/RNA Synthesis |
| Pefloxacin Mesylate Dihydrate | Topoisomerase |
| Sodium Phenylbutyrate | HDAC |
| Fidaxomicin | DNA/RNA Synthesis |
| Bergapten | DNA/RNA Synthesis |
| Mechlorethamine HCl | DNA/RNA Synthesis |
| Chloroambucil | DNA/RNA Synthesis |
| Mupirocin | DNA/RNA Synthesis |
| 6-Mercaptopurine (6-MP) Monohydrate | DNA/RNA Synthesis |
| Nitroxoline | Topoisomerase |
| Levofloxacin hydrate | Topoisomerase |
| Picolinamide | PARP |
| Benzamide | PARP |
| Psoralen | DNA/RNA Synthesis |
| 5-methoxyflavone | DNA/RNA Synthesis |
| SN-38 | Topoisomerase |
| Cyclogalegenol | Telomerase |
| Pixantrone Maleate | Topoisomerase |
| 7-Ethylcamptothecin | Topoisomerase |
| Nimustine Hydrochloride | DNA/RNA Synthesis |
| Vidarabine monohydrate | DNA/RNA Synthesis |
| Rimantadine Hydrochloride | DNA/RNA Synthesis |
| Amenamevir | DNA/RNA Synthesis |
| Cytarabine hydrochloride | DNA/RNA Synthesis |
| Cytidine 5′-triphosphate (disodium salt) | DNA/RNA Synthesis |
| AZD2461 | PARP |
| CGK 733 | ATM/ATR |
| NVP-TNKS656 | PARP |
| G007-LK | PARP |
| Beta-Lapachone | Topoisomerase |
| PJ34 HCl | PARP |
| ME0328 | PARP |
| Pyridostatin Trifluoroacetate Salt | DNA/RNA Synthesis |
| E3330 | DNA/RNA Synthesis |
| CRT0044876 | DNA/RNA Synthesis |
| Triapine | DNA/RNA Synthesis |
| Nexturastat A | HDAC |
| (S)-crizotinib | MTH1 |
| Pritelivir (BAY 57-1293) | DNA/RNA Synthesis |
| LMK-235 | HDAC |
| Santacruzamate A (CAY10683) | HDAC |
| CAY10603 | HDAC |
| Niraparib (MK-4827) tosylate | PARP |
| TH287 | MTH1 |
| TH588 | MTH1 |
| BG45 | HDAC |
| BMH-21 | DNA/RNA Synthesis |
| BRD73954 | HDAC |
| NU1025 | PARP |
| SCR7 | DNA/RNA Synthesis |
| 6-Thio-dG | DNA/RNA Synthesis |
| AZ6102 | PPAR |
| SRT2104 (GSK2245840) | Sirtuin |
| CB1954 | DNA alkylator |
| CC-115 | DNA-PK,mTOR |
| Bromodeoxyuridine (BrdU) | DNA/RNA Synthesis |
| Favipiravir (T-705) | DNA/RNA Synthesis |
| VE-821 | ATM/ATR |
| UPF 1069 | PARP |
| Scriptaid | HDAC |
| Tubercidin | DNA/RNA Synthesis |
| Mirin | ATM/ATR |
| RHPS 4 methosulfate | Telomerase |
| Halofuginone | DNA/RNA Synthesis |
| Mitomycin C | DNA/RNA Synthesis |
| Thiomyristoyl | Sirtuin |
| Melphalan | DNA alkylator |
| SRT2183 | Sirtuin |
| Saccharin 1-methylimidazole (SMI) | DNA/RNA Synthesis |
| CeMMEC1 HCl | DNA/RNA Synthesis |
| CeMMEC13 | DNA/RNA Synthesis |
| NMS-P118 | PARP |
| BGP-15 2HCl | PARP |
| Rbin-1 | DNA/RNA Synthesis |
| YU238259 | DNA-PK |
| E7449 | PARP |
| LTURM34 | DNA-PK |
| B02 | DNA/RNA Synthesis |
| Salermide | Sirtuin |
| SRT3025 HCl | Sirtuin |
| TAS-102 | DNA/RNA Synthesis |
| BAY 1895344 (BAY-1895344) | ATM/ATR |
| Koumine | DNA synthesis |
